# A dual action coumarin-camptothecin polymer for light responsive drug release and photodynamic therapy†

**DOI:** 10.1039/d3py01137b

**Published:** 2023-12-05

**Authors:** Paige A. Shaw, Maxime Klausen, Mark Bradley

**Affiliations:** a EaStCHEM School of Chemistry, University of Edinburgh David Brewster Road EH9 3FJ Edinburgh UK; b Department of Materials, Department of Bioengineering, and Institute of Biomedical Engineering, Imperial College London London SW7 2AZ UK m.klausen@imperial.ac.uk; c Precision Healthcare University Research Institute, Queen Mary University of London Empire House London E1 1HH UK m.bradley@qmul.ac.uk

## Abstract

A light-responsive polymer allowing the controlled release of camptothecin and the generation of reactive oxygen species (ROS) is reported. The polymer was prepared by controlled copolymerisation of water-soluble *N*,*N*-dimethyl acrylamide with a bromocoumarin methacrylate monomer. The lipophilic chemotherapy agent camptothecin was caged onto the coumarin unit *via* a photo-cleavable carbonate ester enabling light-triggered cargo release. The polymer showed good biocompatibility in the dark, and high cancer cell killing activity mediated both by the photo-release of camptothecin and ROS generation.

## Abstract 2

A light-responsive polymer caging the anticancer drug camptothecin was prepared by RAFT polymerisation. The water-soluble polymer efficiently killed cancer cells under irradiation thanks to simultaneous camptothecin uncaging and photodynamic therapy.

## Graphical abstract

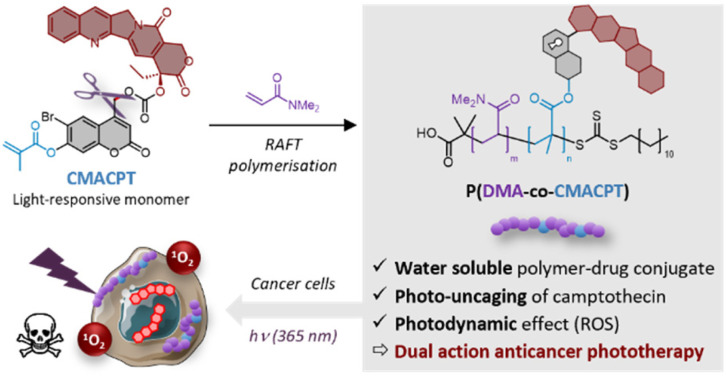

Polymer-based, drug-delivery systems have revolutionised therapeutic strategies by providing solutions to the limitations of many active pharmaceutical ingredients.^1^ Strategies of encapsulation,^2^ and chemical modification^3^ of drugs have successfully improved the solubility, bioavailability and circulation time of many drugs, with several clinically approved polymeric formulations.^4^ Polymer-based drug-delivery systems not only have the ability to improve the pharmacokinetics properties of existing drugs, but also have the ability to rescue compounds that failed clinical development on account of poor solubilities, high toxicities or poor therapeutic indices.^5^

The covalent attachment of drugs onto a polymeric backbone is a particularly versatile strategy, as it leads to fine-tuned bioavailability, metabolism and drug loading, but also enables the incorporation of targeting and triggering elements to deliver therapeutic agents to specific locations. Incorporating stimulus-responsive monomers within these smart co-polymers enables controlled drug-release patterns based on internal or external triggers.^6–8^ In this field, light-activated polymers are of particular interest as they combine these benefits with spatially and temporally controlled drug delivery.^8,9^ Light can be used to trigger a variety of effects on polymer vehicles, such as morphological changes,^10^ crosslinking,^11^ bond cleavage,^12,13^ or oxidation *via* reactive oxygen species (ROS) generation.^14^ Light-responsive polymers can exploit and combine these phenomena for multimodal therapeutic strategies.^7,15^ In cancer, where the complex nature of tumours decreases the efficiency of single-component therapies, the ability to simultaneously combine the effects of chemo- and photo-dynamic therapy (PDT) is attractive, and has shown promising results.^16–19^

Here we targeted the development of a polymeric construct where drug release and ROS generation were promoted by a single light-responsive monomer acting both as a photocleavable protecting group^20^ and a photosensitizer.^21^ Camptothecin (CPT), a natural alkaloid targeting type-I topisomerase that is overexpressed in many cancers,^22^ was selected here as the caged pharmaceutical ingredient. Due to its poor water solubility and bioavailability, combined with high levels of toxicity, camptothecin cannot be administered clinically and is a prime candidate for drug repositioning,^23^ particularly *via* covalent caging strategies that render camptothecin inactive.^19,24–26^ Therefore, Camptothecin was previously incorporated within various drug delivery systems,^27^ including polymer–drug conjugates MAG-CPT (*N*-(hydroxypropyl) methacrylamide),^28^ CT-2106 (poly-l-glutamate),^29^ Pegamotecan (polyethylene glycol),^30^ XMT-1001 (poly(1-hydroxymethylethylene hydroxymethylformal))^31^ and CRLX101 (cyclodextrin-based polymer)^32^ that were all used in clinical trials. Whilst these systems focused on enhanced biodistribution and sustained release, we hereby introduced an external light stimulus to trigger release, and selected for this purpose the coumarin-4-ylmethyl photocleavable unit that has been widely used for uncaging of bioactive substances, including in polymers and nano-systems.^33–36^ The 6-bromo-7-hydroxycoumarin-4-ylmethyl (BHC) unit^33^ was initially modified to incorporate a methacrylate polymerizable unit on its phenol as an ester bond at the 7-position of this coumarin methacrylate (CMA) had been reported as being photo-cleavable.^37^ However, this bond proved to be unaffected by light (see ESI†). Instead, camptothecin was caged by attachment onto the known light-cleavable 4-position of the coumarinylmethyl unit *via* a carbonate group. This gave rise to a camptothecin-based light-activatable monomer (here abbreviated CMACPT) able to ‘switch on’ the chemotherapeutic properties of the drug, while the bromo functionality contributed to the efficient generation of ROS thanks to the heavy atom effect.^38^ This difunctional building block was integrated into a poly(*N*,*N*-dimethyl acrylamide) (PDMA) backbone, yielding a water-soluble, bifunctional, polymeric therapeutic delivery system P(DMA-*co*-CMACPT), and this co-polymer's therapeutic potential was validated *in vitro* showing high cell killing abilities.

The initially targeted 6-bromo-7-hydroxycoumarin-4-ylmethyl scaffold was synthesised by Pechmann condensation between 4-bromoresorcinol 1 and ethyl 4-chloroacetoacetate 2 (Scheme 1).^33^ The chloromethyl intermediate 3 was obtained in a 72% yield, and hydrolysed to the hydroxymethyl derivative using a mixture of hydrochloric acid in DMF. The phenol group of coumarin 4 was preferentially functionalised by the slow addition of methacryloyl chloride under basic conditions, yielding the monofunctionalised coumarin monomer **CMA** in moderate yield (38%). The reported photosensitivity of this monomer^37^ was however disproven in a series of irradiation experiments where no ester photo-cleavage was observed (see ESI†) following extensive photolytic studies. The absence of photosensitivity of the 7-position of the coumarinylmethyl scaffold is consistent with its electronic distribution and associated reactivity.^39,40^ On the contrary, the electron donation coming from the 7-position increases the antibonding contribution on the 4-hydroxymethyl group *via* Zimmermann *ortho*–*meta* donation in the excited state, leading to excited state solvolysis at the 4-hydroxymethyl position,^39,40^ and this was exploited here.

**Scheme 1:**
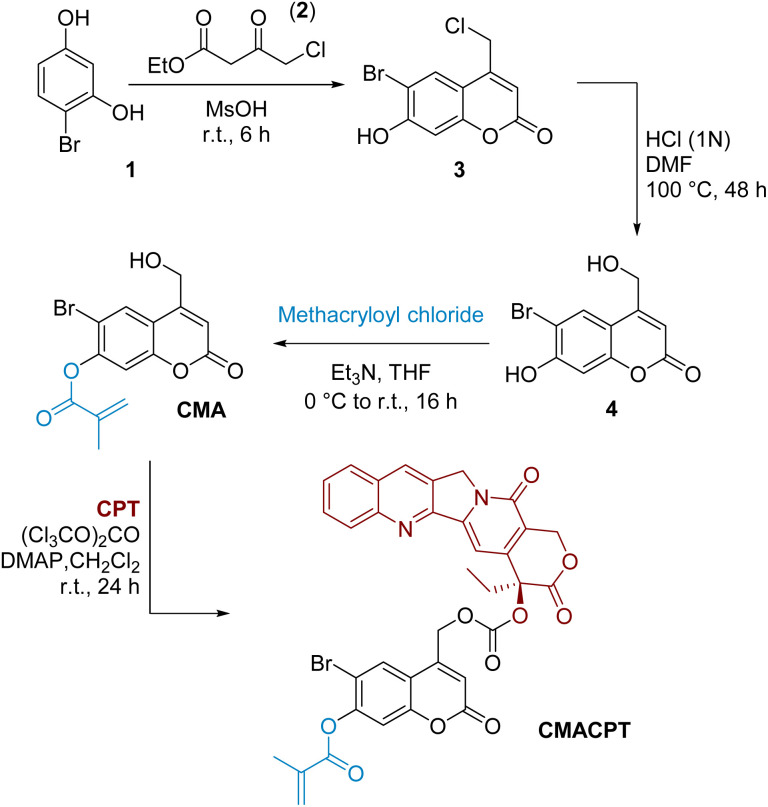
Synthesis of the bifunctional monomer (CMACPT) containing caged camptothecin and a polymerisable methacrylate unit.

The highly toxic, hydrophobic, anti-cancer drug camptothecin was conjugated onto the photoactive 4-hydroxymethyl position *via* its tertiary hydroxyl group in a one-pot procedure using triphosgene as the activated carbonate source.^24^ This gave the photo-sensitive bifunctional monomer CMACPT in good yield (see ESI† for NMR and MS data). This monomer was then co-polymerised with *N*,*N*-dimethylacrylamide (DMA) to yield a water-soluble (>2 mg mL^−1^) random co-polymer P(DMA-*co*-CMACPT) containing the caged drug on its side chain. A DMA : CMACPT monomer ratio of 1 : 50 was selected to include enough DMA to ensure solubility in biological media, while providing sufficient drug levels (considering the IC50 of camptothecin on HeLa cells is 0.4 μM).^41^

Copolymerisation of the bifunctional monomer CMACPT and DMA was performed *via* Reversible Addition–Fragmentation chain-Transfer (RAFT) in dioxane : D_2_O (90 : 10, v : v) using 2-(dodecylthiocarbonothioylthio)-2-methylpropionic acid (DDMAT) as the chain-transfer agent and AIBN as the initiator. Consistent with the data available for this commercially available RAFT agent, the methacrylate monomer polymerised slightly faster than DMA, which may lead to the CMACPT units being closer together within the random copolymer chains. Highly size-controlled (∼12 kDa) and low PDI polymers were obtained and fully characterised (Table 1). Integration of the ^1^H NMR spectrum of the polymer also confirmed the average incorporation of 1 CMACPT per 50 DMA units on the chain, in accordance with the ratio of reagents used in the polymerisation (see ESI†).

**Table 1:** Synthesis and characterisation of the CPT-delivery polymer P(DMA-*co*-CMACPT) *via* RAFT polymerisation

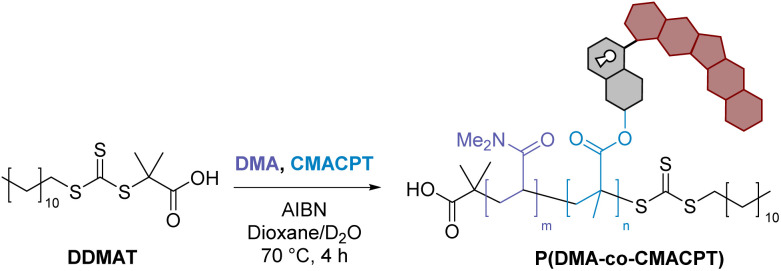	
P(DMA-*co*-CMACPT)	
Monomer Conv.^a^ [%]	99
Theor. size^b^ [%]	11.6
*M*_w_ [kDa] (^1^H NMR)	12.8
*M*_w _ c [kDa] (GPC)	12
PDI	1.29

a Monomer conversion determined by ^1^H NMR.

b Based on monomer conversion and the mass of the RAFT agent added.

c Determined by GPC using DMF with 0.1% LiBr as eluent and PMMA as reference standards.

The bifunctional monomer CMACPT had the expected absorption band at 350 nm (ESI, Fig. S1†)^33^ and under 365 nm irradiation released camptothecin, after photocleavage and decarboxylation (ESI, Scheme S1†). The kinetics of uncaging and efficiency were determined *via* irradiation with a 365 nm light source (Fig. S2†) and HPLC analysis of a solution of the bifunctional monomer CMACPT (Fig. 1a). During the course of the irradiation, the peak for CMACPT (starting concentration of 100 μM) decreased, giving complete and quantitative CPT release after 60 seconds (Fig. 1b). Kinetic analysis confirmed a first order reaction, from which the quantum yield of the uncaging reaction (*Φ*_u_) relative to an actinometry reference (see ESI, Fig. S3†) was determined. A *Φ*_u_ value of 2.3% was determined, consistent with the unmodified BHC photocleavable group (*Φ*_u_ = 1.9% for photo-release *via* a carbamate bond).^33^ The effect of the bromine on the excited state of the coumarin, with a possible triplet state contribution promoted by its heavy-atom character, was then investigated.

**Fig. 1:**
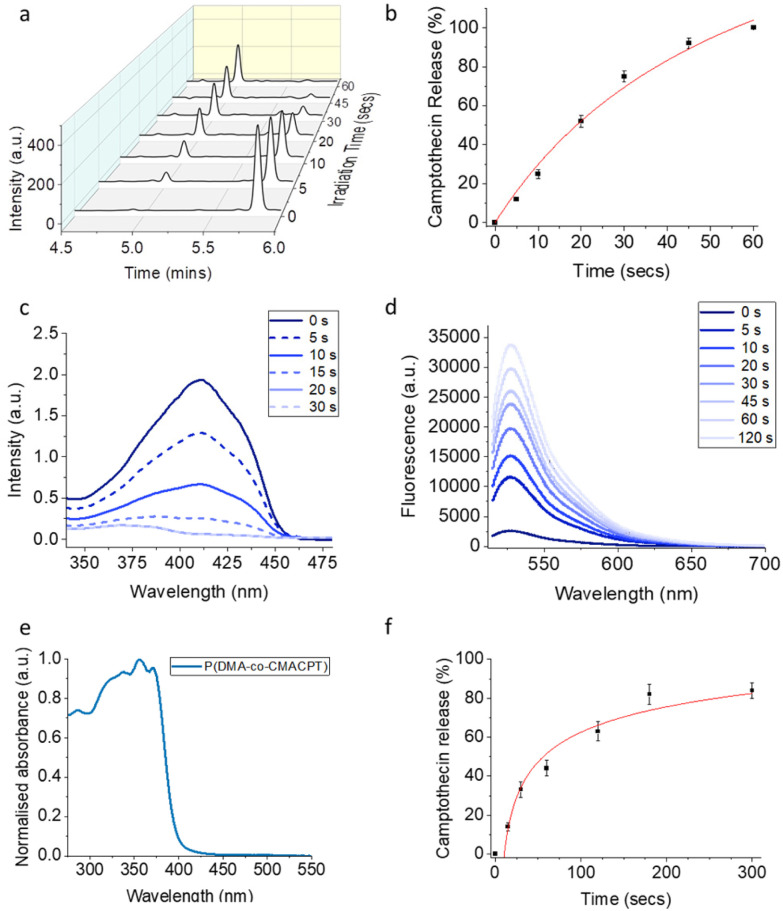
(a) Evolution over time of the HPLC traces (detection 310 nm) of the monomer CMACPT during 365 nm irradiation in a 50 : 50 mixture of H_2_O : acetonitrile *t*_R_ (CMACPT) = 5.82 min, *t*_R_ (CPT) = 5.02 min; (b) drug release over time under irradiation (365 nm) (SD determined from triplicate measurements); (c) evolution of the absorption spectrum of a solution of ^1^O_2_ sensor DPBF (50 μM) and CMACPT (5 μM) in acetonitrile upon excitation at 365 nm; (d) evolution of the fluorescence spectrum of a solution of the ROS sensor DHR123 (5 μM) and CMACPT (5 μM) in acetonitrile upon excitation at 365 nm; (e) normalised absorption spectrum of the P(DMA-*co*-CMACPT) polymer in PBS; (f) kinetic profile of CPT release from the polymer during irradiation (365 nm) in PBS. Values are mean ± SD.

Solutions of the monomer were irradiated in the presence of the singlet oxygen sensor 1,3-diphenylisobenzofuran (DPBF) or the ROS sensor dihydrorhodamine-123 (DHR123), and the evolution of the absorption and fluorescence spectra were respectively monitored (Fig. 1c and d). The 6-bromo-7-hydroxycoumarin methacrylate proved very efficient at generating both ^1^O_2_ and other ROS, as attested by a singlet oxygen quantum yield (*Φ*_Δ_) of 44% (see ESI†). This therefore confers a dual photo-therapeutic mechanism with drug delivery and PDT. It also supports the possible contribution of triplet-state reactivity in the photocleavage mechanism of BHC.^33,42^

The incorporation of the coumarin-CPT monomer onto the polymer backbone was analysed by UV-Vis spectroscopy in PBS (Fig. 1e), and showed the presence of a large absorption band characteristic of the coumarin caging group and the CPT chromophore peaking at 356 nm and tailing beyond 400 nm, indicating a slight bathochromic shift compared to the monomer. To verify that light sensitivity was preserved in the polymer, the photolysis of P(DMA-*co*-CMACPT) (2.0 mg mL^−1^, 365 nm) was analysed by LCMS (ESI, Fig. S4†). The initial HPLC trace showed a broad peak at 5.33 minutes corresponding to the CPT conjugated to the polymer. During the course of the irradiation a peak corresponding to the released CPT appeared (4.75 min, *m*/*z* = 349.9, ESI, Fig. S5†) and increased with irradiation time (Fig. 1f). Quantification of the cleavage rate, determined by looking at the difference in peak intensity between polymer-caged CPT and released CPT, combined with a CPT calibration curve (ESI, Fig. S6†) which confirmed that 82% of the caged CPT was released from the polymer after 3 minutes of irradiation (Fig. 1f). Thus, an irradiation time of 2 minutes was selected in our biology assays to optimise the cleavage while minimising chances of cell damage under 365 nm irradiation.

To validate the photo-therapeutic potential of the P(DMA-*co*-CMACPT) polymer HeLa cells were incubated with the polymer in complete media overnight (6.75–250 μg mL^−1^ – corresponding to concentrations of CPT of 0.375 μM to 15 μM, respectively). Cells were then washed and irradiated at 365 nm for 2 minutes, or maintained in the dark as a control (with the washing step removing the excess polymer, and therefore ensuring that only the internalised polymer generates camptothecin and ROS). The resulting cell toxicity was quantified by an MTT assays 2, 4 and 24 hours post-irradiation (Fig. 2, and ESI, Fig. S9†) in order to distinguish immediate and longitudinal cytotoxicity.

**Fig. 2:**
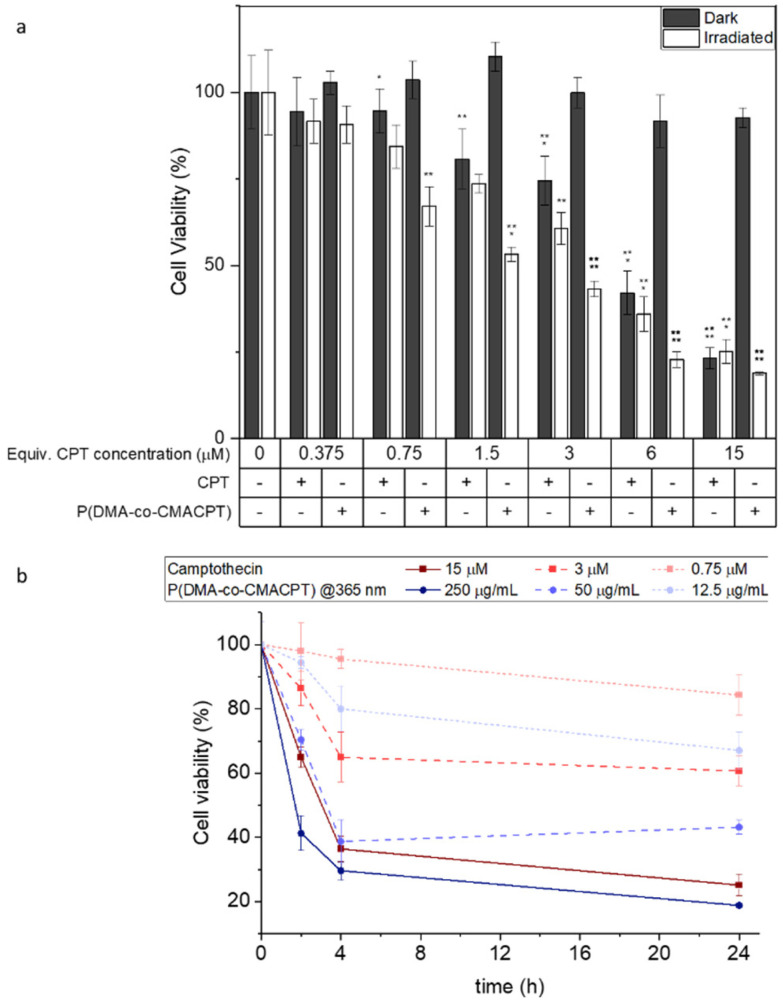
(a) Cell viability 4 h post-treatment of HeLa cells (MTT assay) following exposure to varying concentrations of P(DMA-*co*-CMACPT) (6.75–250 μg mL^−1^, corresponding to equivalent concentrations of camptothecin of 0.375 μM to 15 μM) kept either in the dark or following a 2-minute 365 nm irradiation. Values are mean ± SD, *n* = 3, ** = *p* ≤ 0.01, *** = *p* ≤ 0.001, **** = *p* ≤ 0.0001. (b) Evolution of the cell viability post-treatment (365 nm illumination, 2 min) of HeLa cells treated with P(DMA-*co*-CMACPT) (blue circles) or equivalent concentrations of camptothecin (red squares). Values are mean ± SD.

In all assays, P(DMA-*co*-CMACPT) showed no toxicity in the dark, validating the innocuity of the caged CPT. However, high levels of toxicity were seen on the irradiated cells, even 2 h post-treatment, confirming polymer uptake and validating it as a selectively switched-on drug delivery system (Fig. S9a†) in a concentration dependent manner. Increasing the post-treatment incubation time to 4 h (Fig. 2a) and 24 h (Fig. S9b†) progressively increased the level of cell death, presumably due to increased exposure to CPT (Fig. 2b), whilst the difference between illuminated samples and the CPT control decreased. After 4 hours, both the 100 μg mL^−1^ and 250 μg mL^−1^ polymer concentrations showed similar levels of toxicity, likely due to drug saturation in the cell death pathway. Cellular morphology, imaged under bright field microscopy, was in accordance with the MTT assay observations (ESI, Fig. S10†). Control samples, irradiated cells, and cells containing the highest concentration of the polymer and kept in the dark, all showed a similar healthy, elongated morphologies at various stages of mitosis (Fig. S10a–c†). Irradiated samples containing polymer showed differing levels of cell death (Fig. S10d–f†), as indicated by the darker rounded cells, with uneven membranes and porous bubble-like contents indicative of cell-death pathways.

In conclusion a bifunctional scaffold based on the 6-bromo-7-hydroxycoumarin-4-ylmethyl uncaging unit was prepared, incorporating a polymerisation handle and a photocleavable caged drug, while also displaying singlet oxygen generation capability. The previously reported photosensitivity of this coumarin at its 7-position was disproven, with the photosensitive 4-position used to cage the drug CPT, to allow its repurposing. The monomer was copolymerized with *N*,*N*-dimethylacrylamide under RAFT conditions to fabricate a fully water soluble, light sensitive, drug delivery polymer P(DMA-*co*-CMACPT). Photo-release of camptothecin from the polymer P(DMA-*co*-CMACPT) led to 82% cleavage with 3 minutes of irradiation. Additionally, efficient ROS generation character was evidenced with the bromocoumarin caging units, which is consistent with previous reports evidencing triplet-state contribution to their photo-cleavage, and leveraging the heavy-atom effect on halogenated hydroxycoumarin derivatives.^33,42^ The dual phototherapeutic potential of the P(DMA-*co*-CMACPT) was confirmed with highly effective cell killing observed *via* combined ROS and camptothecin release. Cell-viability analysis suggested that ROS generation might be responsible for a significant proportion of cell death on a short time frame, whereas the action of the released CPT takes longer to manifest. Indeed, bright field microscopy indicated that different cell-death mechanisms, such as apoptosis, and pyroptosis which was recently proposed as a cell death mechanism under ROS action, may be operational.^43^ Although detailed investigation of cell-death pathways are necessary, this could improve the potential of this combined therapy to tackle resistant cancers and offer a novel dual strategy, with both therapies linked proximally to ensure killing. This work demonstrates the versatility of light-activated polymers with photo-therapy, and also illustrates their potential to enhance the solubility and bioavailability of chemotherapy drugs that are otherwise difficult to formulate or show poor therapeutic indices. Such polymeric drug delivery systems could be tools in drug repositioning and combined targeted therapeutic strategies. Additionally, access to the near-infrared “biological windows”, where tissue penetration is the highest and photo-toxicity the lowest, can be facilitated by the use of the two-photon excitation technique – in which two photons of double the wavelenght (half the energy) are simultaneously absorbed. Since photocleavable protecting groups of the coumarin family (including BHC) are known to show high two-photon absorption cross-sections and photosensitivity,^33,36,44^ this would enable efficient near-infrared activation of the photo-therapeutic effect of such coumarin-containing polymers.

## Conflicts of interest

There are no conflicts to declare.

## Supplementary Material

PY-015-D3PY01137B-s001
