# Supplementary material for: A dual action coumarin-camptothecin polymer for light responsive drug release and photodynamic therapy
Source: Polym Chem. 2023 Dec 5;15(2):54–8. doi: 10.1039/d3py01137b (PMC10758926; doi:10.1039/d3py01137b)
Supplement: PY-015-D3PY01137B-s001 [file PY-015-D3PY01137B-s001.pdf]

Electronic Supplementary Material (ESI) for Polymer Chemistry.
This journal is © The Royal Society of Chemistry 2023

A Dual Action Coumarin Camptothecin Polymer for Light Responsive Drug Release and Photodynamic Therapy

Paige A. Shaw,[a] Maxime Klausen,[a,b*] and Mark Bradley[a, c*]

a. EaStCHEM School of Chemistry, University of Edinburgh, David Brewster Road, EH9 3FJ, Edinburgh, UK.

b. Department of Materials, Department of Bioengineering, and Institute of Biomedical Engineering, Imperial College London, London, SW7 2AZ UK. Corresponding author email: m.klausen@imperial.ac.uk

c. Precision Healthcare University Research Institute, Queen Mary University of London, Empire House, London, E1 1HH, UK. Corresponding author email: m.bradley@qmul.ac.uk

SUPPORTING INFORMATION

Table of content

I. Synthesis and chemical characterization 2
1. Materials and methods 2
2. Synthesis of the CMACPT monomer 3
3. Procedure for RAFT polymerisation 6
II. Photophysical and photochemical characterization 6
1. Materials and methods 6
2. Additional absorption data 7
3. Photolysis and uncaging quantum yield of the CMACPT monomer 7
4. Photolysis of the P(DMA-co-CMACPT) polymer 10
5. Singlet oxygen generation 12
6. Reactive oxygen species generation 14
III. Biological Studies 15
1. Cell Culture 15
2. Cell Viability Assay 15
3. Intra-Cellular Drug Release Studies 16
4. Live Cell Microscopy 17
IV. Investigations of the behaviour of CMA under irradiation 19
1. Synthesis of the P(DMA-co-CMA) random copolymer 19
2. Irradiation of the CMA monomer 19
3. Irradiation of the P(DMA-co-CMA) random polymer: NMR follow-up 21
4. Irradiation of the P(DMA-co-CMA) random polymer: absorption follow-up 22
V. NMR, MS and HPLC data 24
VI. Additional references 30

I. Synthesis and chemical characterization

1. Materials and methods

All air and moisture sensitive manipulations were carried out using standard techniques, with oven-dried reaction vessels, anhydrous solvents, and under nitrogen atmosphere. Extraction and column chromatography solvents were purchased in anhydrous form, and used as received. All reagents were purchased from Fisher Scientific, Aldrich or Fluorochem and used without further purification unless indicated otherwise. AIBN was recrystallised from MeOH and *N*,*N*-dimethyl acrylamide (DMA) was filtered through a plug of basic alumina to remove inhibitors prior to use. Thin layer chromatography (TLC) was performed on Merck silica gel 60 F254 aluminum plaques, and column chromatography was performed on Macherey-Nagel silica gel 60 (40-63 µm). Reverse phase flash chromatography was performed on a Biotage® Isolera One purification system using Biotage® SNAP Ultra C18 columns.

^{1}H and ^{13}C spectra were recorded on a Bruker AVA 500 spectrometer at 500 MHz and 126 MHz, or on a Bruker AVA 600 spectrometer at 600 MHz and 151 MHz respectively. Shifts (δ) are given in parts per million with respect to solvent residual peak, and coupling constant (J) are given in Hertz.

Analytical reverse-phase high-performance liquid chromatography (RP-HPLC) was performed on an Agilent 1100 system equipped with a Kinetex XB-C18 column (50 × 4.6 mm, 5 µm) with a flow rate of 1 mL/min. Samples were eluted either with a gradient of H_2O/MeCN 95/5 to 5/95, all buffered with 0.1% formic acid, over 6 min, then holding at 95% for 3 min, followed by elution at 5% ACN. Detection was performed with a multiple wavelength detector (MWD) at 254, 282, 310 and/or 365 nm, and by an evaporative light scattering (ELSD) detector. GPC was performed on an Agilent 1100 GPC equipped with PLgel MIXED-C columns (2 × 102 – 2 × 106 g/mol, 5 mm) and an RI detector, eluting with DMF containing 0.1 % w/v LiBr at 60 °C and a flow rate of 1 mL/min. Polymer solutions unless specified otherwise were dissolved at concentrations of 5 mg/ml in DMF containing 0.1 % LiBr. Samples were run at 60 °C for 30 minutes at flow rate of 1 mL/min.

Low resolution electrospray ionization mass spectrometry (ESI-MS) analyses were carried out on an Agilent Technologies LC/MSD Series 1100 quadrupole mass spectrometer (QMS) in ESI mode. HR-MS were obtained by the Mass Spectrometry department of the University of Edinburgh and were performed on a Finnigan MAT 900 XLP high resolution double-focusing mass spectrometer. MALDI

spectra were acquired on a Bruker Ultraflextreme MALDI TOF/TOF with a matrix solution of sinapinic acid (10 mg/mL) in $H_2O/CH_3CN/TFA$ (50/50/0.1).

2. Synthesis of the CMACPT monomer

7-hydroxy-6-bromo-4-chloromethylcoumarin (3)[1]

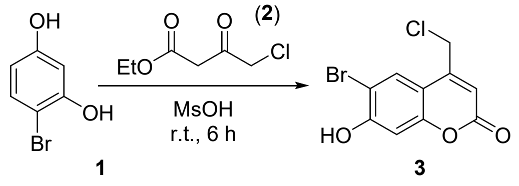

A solution of 4-bromoresorcinol (**1**) (1 g, 5.29 mmol, 1 equiv.) and methanesulfonic acid (8 ml) was stirred for 15 minutes until the solid dissolved. Ethyl 4-chloroacetoacetate (**2**) (0.92 ml, 7.94 mmol, 1.5 equiv.) was added to the solution and stirred for a further 6 hours. Iced-water (24 ml) was added to the resulting mixture and stirred for 30 minutes to yield a white precipitate. The precipitate was collected via filtration and recrystallised in hot EtOH (17 ml). The final product was collected via filtration.

Yield 72 % (1.1 g white solid). **^{1}H NMR (500 MHz, DMSO-*d*$_6$) δ (ppm):** 11.54 (1H, *br*, s), 8.00 (1H, s), 6.92 (1H, s), 6.48 (^{1}H, t, *J* = 1.5 Hz), 5.00 (2H, d, *J* = 0.7 Hz). **^{13}C NMR (126 MHz, DMSO-*d*$_6$) δ (ppm):** 159.6, 157.5, 154.1, 150.1, 129.0, 112.1, 110.7, 106.2, 103.3, 41.2. **LCMS (ESI)** *m/z* 289.0 $(M+H)^+$. **HPLC (282 nm detection)** t_R = 4.546 min.

6-Bromo-4-(chloromethyl)-7-hydroxycoumarin (4)

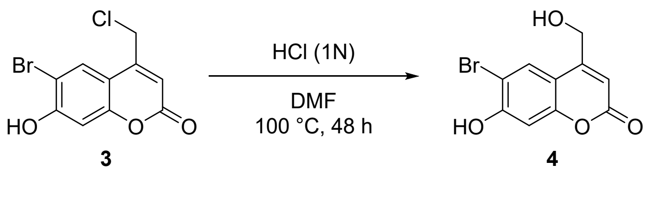

Compound **3** (2 g, 7.38 mmol, 1 equiv.), was dissolved in a mixture of DMF (60 mL) and 1N HCl (30 mL) and heated to 100 °C for 48 h. Following cooling, the solvent was evaporated under reduced pressure. The product was resuspended in EtOAc (100 mL) and washed in brine (3 × 50 mL) and H_2O (3 × 50 mL). The organic layer was dried with $MgSO_4$, filtered and dried *in vacuo* to give **4**.

Yield 67% (light yellow solid). **^{1}H NMR (500 MHz, DMSO-*d_6*) δ (ppm)**: 11.38 (1H, *br*, s), 7.84 (1H, s), 6.89 (1H, s), 6.27 (1H, t, *J* = 1.5 Hz), 5.60 (1H, t, *J* = 5.6 Hz), 4.69 (2H, dd, *J* = 5.8, 1.5 Hz). **^{13}C NMR (126 MHz, DMSO-*d_6*) δ (ppm):** 160.6, 157.6, 156.5, 154.2, 128.7, 111.5, 108.1, 106.5, 103.6, 59.6. **LCMS (ESI)** *m/z* 271.0 $(M+H)^+$. **HPLC (282 nm detection)** t_R = 3.285 min.

6-Bromo-4-hydroxymethylcoumarin-7-yl methacrylate (CMA)

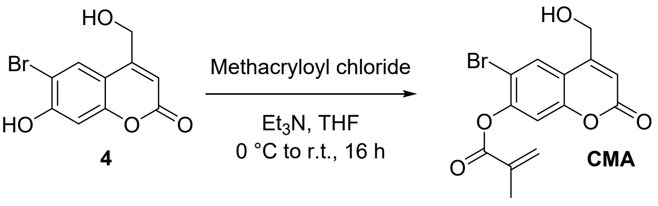

Compound **4** (1 g, 3.7 mmol, 1 equiv.) and Et_3N (0.5 mL, 3.7 mmol, 1 equiv.) were dissolved in THF (84 mL) under a nitrogen atmosphere. A solution of distilled methacryloyl chloride (0.37 mL, 3.7 mmol, 1 equiv.) in THF (16.7 mL) was then added dropwise to the flask at 0 °C and left to warm to room temperature overnight. The resulting insoluble salt was removed by filtration and the solvent was evaporated under reduced pressure. The crude product was purified by column chromatography of silicagel (eluent: CH_2Cl_2/EtOAc, 9/1 to 8/2) to give **CMA**.

Yield 38 % (457 mg white solid). **^{1}H NMR (500 MHz, DMSO-*d_6*) δ (ppm):** 8.07 (s, 1H), 7.59 (s, 1H), 6.51 (t, *J* = 1.6 Hz, 1H), 6.39 (p, *J* = 1.0 Hz, 1H), 6.02 (p, *J* = 1.5 Hz, 1H), 5.73 (t, *J* = 5.5 Hz, 1H), 4.78 (dd, *J* = 5.5, 1.7 Hz, 2H), 2.05 (t, *J* = 1.3 Hz, 3H). **^{13}C NMR (126 MHz, DMSO-*d_6*) δ (ppm):** 164.0, 159.4, 155.4, 152.8, 149.8, 134.4, 129.2, 128.34, 112.7, 111.3, 111.1, 59.1, 17.9. **HRMS (ESI)** *m/z* 338.9874, calculated for $C_{14}H_{12}Br_1O_5$ $(M+H)^+$ 338.98626. **HPLC (282 nm detection)** t_R = 4.650 min.

(*S*)-6-bromo-4-(((((4-ethyl-3,14-dioxo-3,4,12,14-tetrahydro-1*H*-pyrano[3',4':6,7]indolizino[1,2-*b*]quinolin-4-yl)oxy)carbonyl)oxy)methyl)-coumarin-7-yl methacrylate (CMACPT)

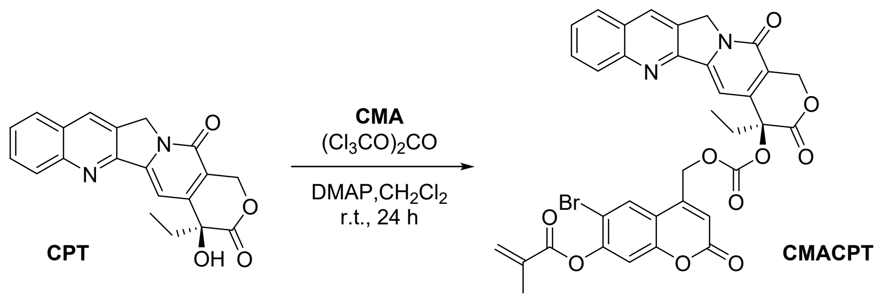

Camptothecin (70 mg, 0.201 mmol, 1 equiv.) and DMAP (73.6 mg, 0.603 mmol, 3 equiv.) were suspended in dry CH_2Cl_2 (2 mL) under argon atmosphere. Triphosgene (20.3 mg, 68.3 μmol, 0.34 equiv.) was then added in one portion and the mixture was stirred for 10 min at room temperature. A solution of the hydroxycoumarin monomer **CMA** (68.1 mg, 0.201 mmol, 1 equiv.) in dry CH_2Cl_2 (2 mL) was then added dropwise over 5 min. The reaction mixture was stirred overnight at room temperature, then directly loaded onto a column chromatography of silicagel (gradient eluent: CH_2Cl_2:EtOAc, 1:0 to 6:4) to give the pro-drug monomer as a white powder.

Yield 70% (68 mg, white powder). **^{1}H NMR (601 MHz, $CDCl_3$) δ (ppm)** 8.38 (s, 1H), 8.19 (d, *J* = 8.5 Hz, 1H), 7.91 (d, *J* = 8.1 Hz, 1H), 7.82 (ddd, *J* = 9.6, 6.4, 1.3 Hz, 1H), 7.68 – 7.63 (m, 1H), 7.62 (s, 1H), 7.29 (s, 1H), 7.16 (s, 1H), 6.54 (d, *J* = 1.4 Hz, 1H), 6.39 (d, *J* = 1.5 Hz, 1H), 5.83 (dd, *J* = 2.5, 1.3 Hz, 1H), 5.70 (d, *J* = 17.0 Hz, 1H), 5.39 (d, *J* = 17.1 Hz, 1H), 5.30 – 5.18 (m, 3H), 2.31 (dq, *J* = 14.8, 7.5 Hz, 1H), 2.19 (dq, *J* = 14.9, 7.5 Hz, 1H), 2.05 (d, *J* = 1.4 Hz, 2H), 1.34 – 1.19 (m, 3H), 1.04 (t, *J* = 7.5 Hz, 2H), 0.87 (t, *J* = 7.1 Hz, 2H). **^{13}C NMR (151 MHz, $CDCl_3$) δ (ppm)** 167.0, 164.1, 159.1, 157.3, 153.3, 153.2, 152.2, 150.9, 149.0, 146.9, 146.3, 145.3, 134.9, 131.3, 130.9, 129.8, 129.1, 128.6, 128.32, 128.27, 128.25, 127.6, 120.4, 116.1, 114.5, 113.0, 112.1, 95.6, 79.0, 67.2, 64.8, 50.2, 34.2, 31.9, 22.4, 18.4, 18.3, 14.16, 14.15, 7.8. **HRMS (ESI):** *m/z* 735.05721, calculated for $C_{35}H_{25}BrN_2O_{10}Na$ $(M+Na)^+$: 735.05848. **HPLC (254 nm detection)** t_R = 5.743 min.

3. Procedure for RAFT polymerisation

Random co-polymerization of DMA and CMACPT.

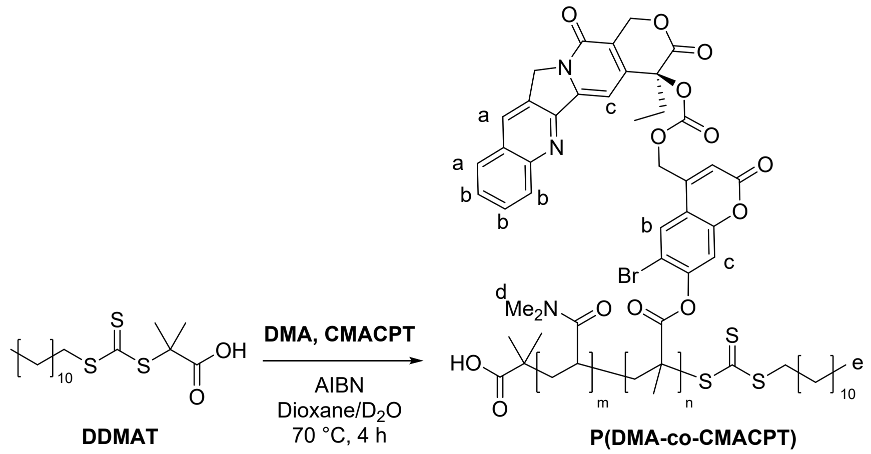

The random copolymer P(DMA-co-CMACPT) was synthesized from the coumarin-camptothecin monomer **CMACPT** and DMA, using 2-(dodecylthiocarbonothioylthio)-2-methylpropionic acid (**DDMAT**) as the RAFT agent and AIBN as the initiator. The polymerisation reactions was performed with a ratio of 2:100:0.2:1 of **CMACPT**:DMA:AIBN:RAFT to prepare a 12 kDa polymer. Briefly, CMACPT (6 mg), DMA (42 µL), DDMAT (1.5 mg) and AIBN (40 µg) were dissolved in a mixture of D_2O:1,4-dioxane (10:90, *v*:*v;* final concentration in monomers ~1 M) in a sealed tube. The mixture was degassed by bubbling argon for 30 min and then heated to 70 °C for 3 h. The polymerizations were then quenched with liquid nitrogen and, after defrosting in air, the polymer was purified by repeated precipitation in Et_2O from MeOH (× 3).

^{1}H NMR (601 MHz, CD_3OD) δ (ppm) 8.75 – 8.31 (H_a), 8.23 – 7.49 (H_b), 7.45 – 7.18 (H_c), 3.08 – 2.82 (H_d), 0.94 – 0.81 (H_e).

II. Photophysical and photochemical characterization

1. Materials and methods

Photophysical studies were performed with freshly prepared air-equilibrated solutions at room temperature (298 K). UV/Vis absorption spectra were recorded on an Agilent 8453 spectrophotometer. Fluorescence measurements were performed on dilute solutions (ca. 10^{-6} M, optical density ≤ 0.1) contained in standard l = 1 cm quartz cuvettes using a Shimadzu RF-6000

spectrofluorometer. The emission spectra were corrected for the wavelength-sensitivity of the detection unit.

2. Additional absorption data

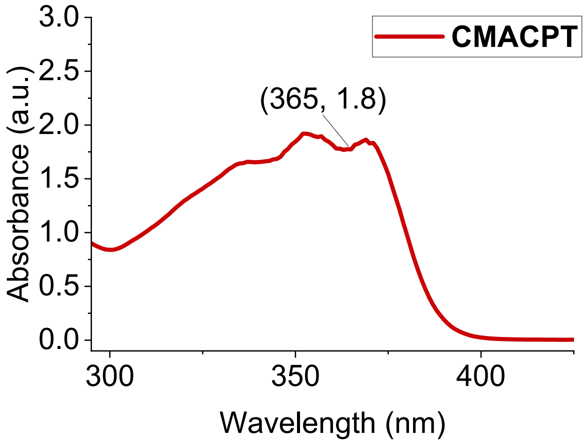

Figure S1: a) Absorbance spectrum of **CMACPT** in MeCN (100 µM). The absorbance value at 365 nm is indicated for reference.

3. Photolysis and uncaging quantum yield of the CMACPT monomer

All light cleavage experiments were performed at room temperature using a UVP CL-1000 Crosslinker (Analytik Jena GmbH, Jena, Germany) equipped with five 8W UV-A tubes (F8T5/BL368; λ_{max} = 365 nm; 5 x 8W, 2 mW cm^{-2}, see Figure S2). A 1.4 mM solution of **CMACPT** was prepared by diluting **CMACPT** (1 mg) in acetonitrile (1 mL). A sample of this stock solution (214 µL) was diluted to 3 mL, with a final ratio of 50:50 H_2O:MeCN (*v*:*v*) to give a concentration of 100 µM. The solution was added to a quartz cuvette, placed within the crosslinker (which allowed a consistent set distance between the sample and the light source), and irradiated for a total of 60 seconds taking aliquots (50 µL) at regular time points. The aliquots were eluted on an HPLC with detection at 310 nm (see materials and methods section). During the course of the irradiation, the peak related to the coumarin monomer **CMACPT** (5.8 min) decreased, while the peak related to the released camptothecin (5.0 min) appeared. The conversion rate (x) was determined by integration of the HPLC peaks, and verified a first order kinetics law (Figure 1b).

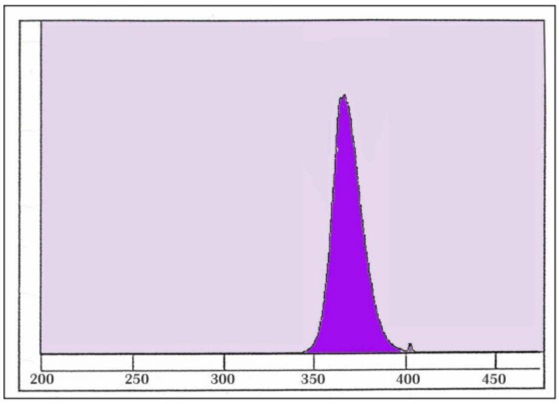

Figure S2. Irradiance spectrum of the UVP CL-1000 Crosslinker light source used in our irradiation experiments, as provided by the supplier.

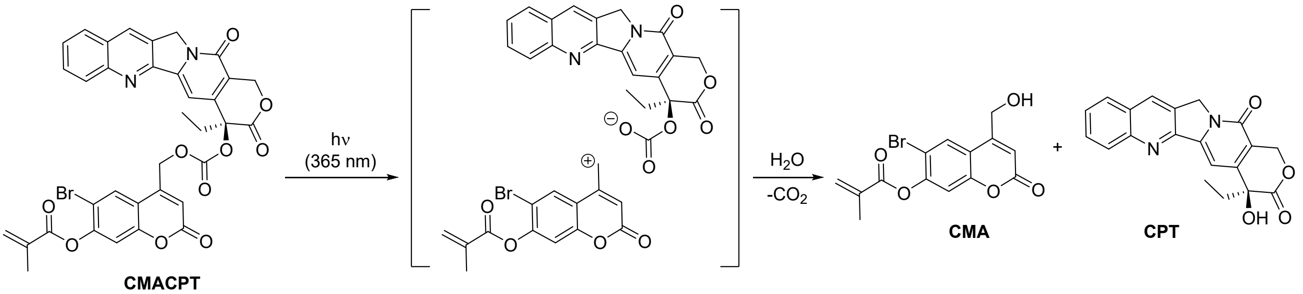

Scheme S1: Photocleavage of coumarin-camptothecin monomer **CMACPT**.

To investigate the efficiency of cleavage a kinetic follow-up was performed to determine the photochemical quantum yield of the uncaging reaction (Φ_u), given by **Equation S1**.

Equation S1: $$\Phi_u = (I\sigma^{\lambda}t_{90\%})^{-1}$$

Where *I* is irradiation intensity expressed in einstein·cm^2·s^{-1}, σ^{λ} is the decadic extinction coefficient at the excitation wavelength λ ($\varepsilon^{\lambda} \times 10^3$, where ε is the extinction coefficient) in cm^2·mol^{-1}, and $t_{90\%}$ is the irradiation time for 90 % photolysis conversion, given in seconds.

The total irradiation intensity *I* at 365 nm was measured using the well-described chemical actinometry method.[2,3] Potassium Ferrioxalate (295 mg) was dissolved in 90 mL deionised water and 10 mL H_2SO_4 (solution 1). Separately, NaOAc·3H_2O (7.35 g), 1,10-phenanthroline·H_2O (30 mg) was dissolved in 20 mL deionised water and 0.9 mL conc. H_2SO_4 (solution 2). Solution 1 (V_1 = 2 mL,

0.006 M) was added to two cuvettes. One cuvette was irradiated for 5 seconds at 365 nm, and the other was kept in the dark. Solution 2 (V_2 = 330 μL) was then added to each cuvette, and the absorbance was measured at 510 nm (Figure S2).

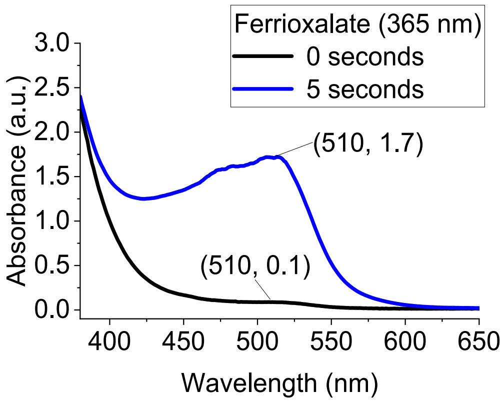

Figure S3. Absorbance spectra of potassium ferrioxalate and 1,10-phenanthroline mixture before and after 5 seconds of 365 nm irradiation.

The number of moles of Fe^{2+} ions generated per unit time of irradiation (n^{Fe2+} in mol·s^{-1}), was determined using Equation S2.

Equation S2:

$$n^{Fe2+} = \frac{(V_1 + V_2) \times \Delta A_{510}}{t \times l \times \varepsilon_{510}}$$

Where ΔA_{510} is the change in absorbance of the Fe^{2+}-phen complex at 510 nm before and after irradiation (ΔA_{510} = 1.6), ε_{510} is the molar absorptivity of the Fe^{2+}-phen complex at 510 nm ($\varepsilon_{510} = 1.1 \times 10^4$ $M^{-1}cm^{-1}$, according to literature[3]), l is the optical path length (cm), t is the time of irradiation of the potassium ferrioxalate solution (t = 5 s), and $V_1 + V_2$ is the total volume of solution ($V_1 + V_2 = 2.33 \times 10^{-3}$ L).

The irradiation intensity *I* of the 365 nm light supply was then calculated using Equation S3.

Equation S3:

$$I = \frac{n^{Fe2+}}{\Phi_{Fe2+}}$$

Where Φ_{Fe2+} is the quantum yield of production of ferrous ions by photoreduction of ferrioxalate at the excitation wavelength. The value reported for photoreduction of a 0.006 M ferrioxalate solution

at 365 nm was used here (Φ_{Fe2+} = 1.21).[3] Total irradiation intensity *I* of the 365 nm light source was calculated to be 5.6 × 10^{-8} einstein·cm^{2}·s^{-1}.

Using the absorption spectrum of **CMACPT** in the photolysis solvent (Figure S1) and the first order kinetic plot allowed determination of the σ_{365} and $t_{90\%}$ parameters respectively (ε_{365} = 1.8 × 10^4 M^{-1} cm^{-1}; $t_{90\%}$ = 45 s). The uncaging quantum yield Φ_u of **CMACPT** was therefore determined to be 2.3 %.

4. Photolysis of the P(DMA-co-CMACPT) polymer

A 2 mg/ml solution of **P(DMA-co-CMACPT)** was prepared in a 50:50 mixture of H_2O (0.1 % formic acid buffer) and Acetonitrile (0.1 % formic acid buffer) The solution was added to a quartz cuvette, placed within the crosslinker and irradiated for a total of 5 minutes taking aliquots (50 µL) at regular time points (0 seconds, 15 seconds, 30 seconds, 45 seconds, 60 seconds, 120 seconds, 180 seconds, 300 seconds of cleavage). The aliquots were eluted on an RP-HPLC coupled to a mass spectrometer in ESI mode scanning the range *m/z* = 100-1000 in positive mode. During the course of the irradiation, the peak height at t_R = 5.33 min decreased, while the peak height at t_R = 4.75 min related to the released camptothecin increased (Figure S3). Calculations of cleavage were based on the assumption that the concentration of camptothecin within the polymer sample (at the 2 mg/mL concentration irradiated) was approximately 0.120 mM based on the composition of the polymer.

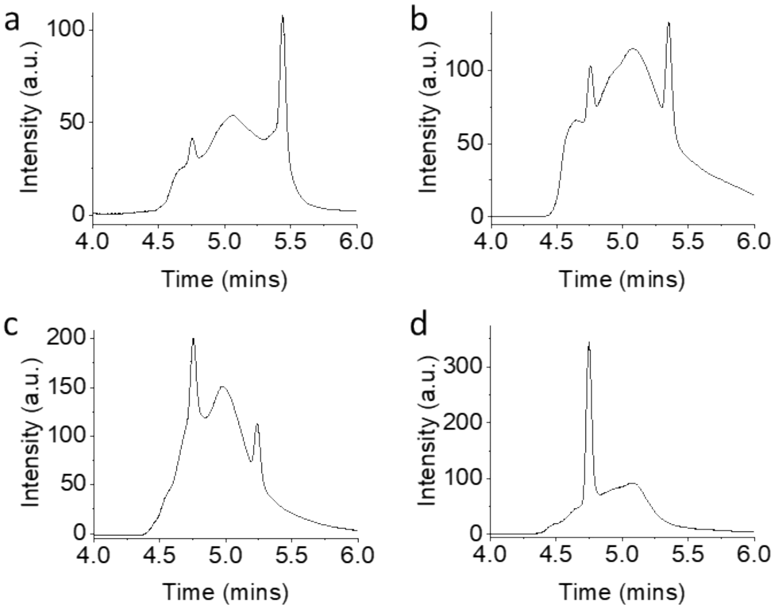

Figure S4: HPLC analysis of the cleavage under 365 nm irradiation of camptothecin from the **P(DMA-co-CMACPT)** polymer (2 mg/mL) in MeCN:H_2O, 50:50 (*v*:*v*). 50 µL polymer samples were taken at a)

t = 0; b) t = 15 s; c) t = 30 s; d) t = 180 s for analysis. Note: the wide peak shape likely reflects the natural distribution of the polymer and the interactions of the chains with the stationary and mobile phase; sample at t = 0 contained no camptothecin (verified by ESI-MS, Figure S5a).

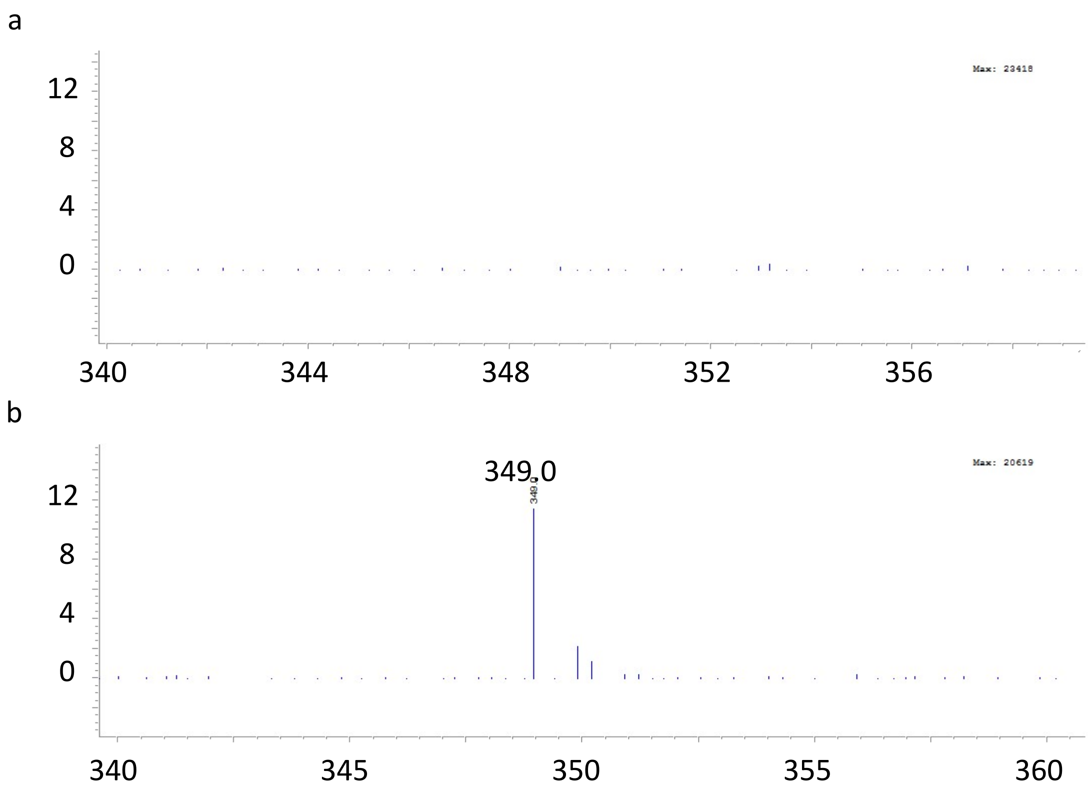

Figure S5. LCMS mass spectra of the **P(DMA-co-CMACPT)** polymer before and after irradiation. Samples ran at 2 mg/mL concentration with 20 µL injection volume eluting with a gradient of H_2O/MeCN, buffered with 0.1% formic acid. Mass spectra were acquired from the region on the spectra corresponding to the **P(DMA-co-CMACPT)** polymer peak (4.75 min). a) t = 0 confirming no free camptothecin was present in the polymer solution prior to irradiation. b) Mass spectrum showing the presence of camptothecin after 15 minutes of irradiation under 365 nm light.

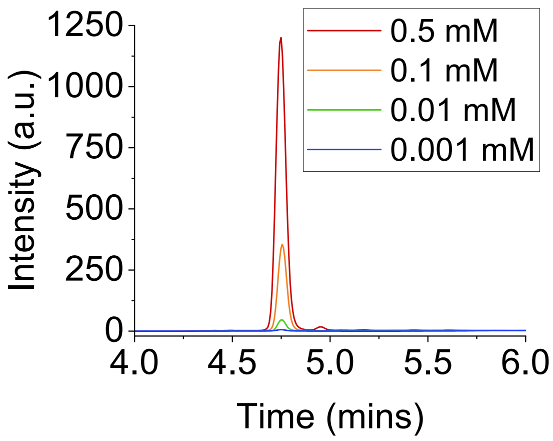

Figure S6. HPLC calibration of camptothecin, eluting with MeCN and H_2O (buffered with 0.1 % formic acid) with 20 μL injection volumes.

5. Singlet oxygen generation

The relative singlet oxygen quantum yield (Φ_Δ) of the coumarin was determined according to the relative method described in literature,[4] using 1,3-Diphenylisobenzofuran (DPBF) as singlet oxygen trap. Air saturated solutions of the monomer (5 μM) and DPBF (50 μM) in acetonitrile were irradiated in a UVP CL-1000 Crosslinker (Analytik Jena GmbH, Jena, Germany) equipped with five 8W UV-A tubes (F8T5/BL368; λ_{max} = 365 nm; 5 x 8W, 2 mW cm^{-2}). During the course of the irradiation (0 to 30 s), the decrease in absorbance at 410 nm (λ^{max} of DPBF) of the sample was monitored over time in order to follow the photo-oxidation reaction occurring between the DPBF sensor and the $^1O_2$ generated (scheme S2). The kinetics of the photo-oxidation reaction for the coumarin monomer was then compared with a reference compound (Benzophenone, Φ_Δ = 39% in acetonitrile[5]) irradiated under identical experimental conditions. The quantum yield was then calculated according to the following equation:

Equation S4:

$$\Phi_\Delta^S = \Phi_\Delta^{ref} \times \left(\frac{m^S}{m^{ref}}\right) \times \frac{1 - 10^{-A^{ref}(\lambda_{irr})}}{1 - 10^{-A^S(\lambda_{irr})}}$$

Where the superscripts *S* and *ref* represent respectively the measured sample and the known reference, m is the slope of the decrease in absorbance (A-A_0) of the $^1O_2$ sensor at 410 nm over time,

and $1 - 10^{-A(\lambda_{irr})}$ is a correction factor taking into account the difference in optical density of the samples at the irradiation wavelength (365 nm).

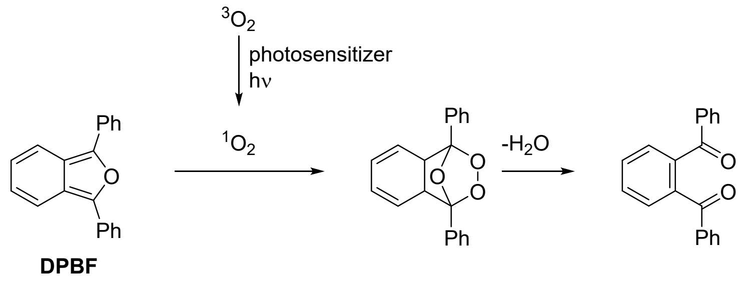

Scheme S2. Photo-oxidation of DPBF by singlet oxygen occurring upon excitation of a photosensitizer.

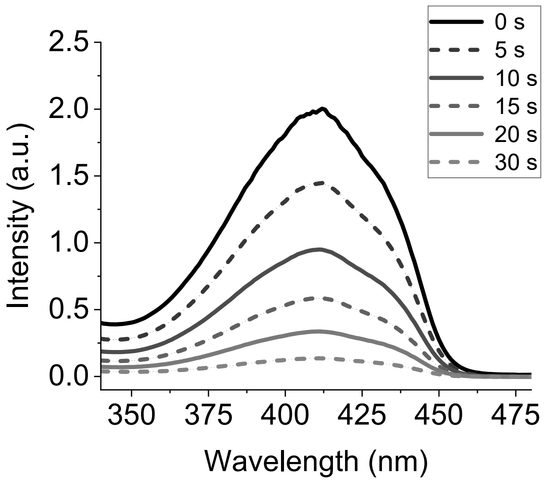

Figure S7. Evolution of the absorption of a solution containing the $^1O_2$ sensor DPBF and reference benzophenone in MeCN upon excitation at 365 nm.

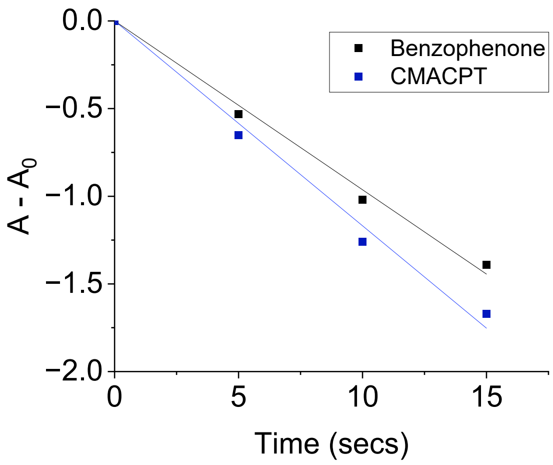

Figure S8. Kinetics of the decrease in absorbance of the DPBF sensor at 410 nm over time during irradiation (365 nm) in the presence of benzophenone (black) and CMACPT (blue).

6. Reactive oxygen species generation

The ability of the coumarin monomer to generate ROS (other than singlet oxygen) upon irradiation was investigated using dihydrorhodamine 123 (DHR 123) as a sensor. Air saturated solutions of the coumarin (5 µM) and DHR123 (5 µM) in water/acetonitrile (1/1, *v*/*v*) were irradiated in a UVP CL-1000 Crosslinker (Analytik Jena GmbH, Jena, Germany) equipped with five 8W UV-A tubes (F8T5/BL368; λ_{max} = 365 nm; 5 x 8W, 2 mW cm^{-2}). During the course of the irradiation, the increase in fluorescence intensity of the samples at 528 nm (λ_{exc} = 500 nm) was monitored over time in order to follow the light mediated oxidation of DHR123 into the fluorescent rhodamine 123 (scheme S3).

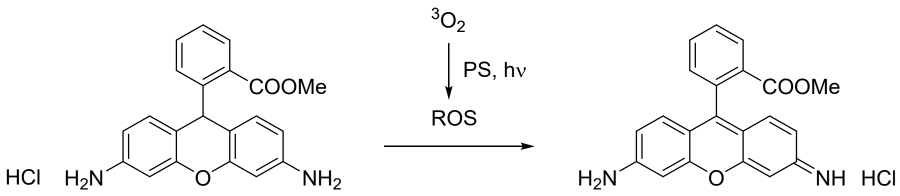

Scheme S3. Light mediated-oxidation of DHR123 into Rhodamine 123 by ROS generated upon excitation of a photosensitizer.

III. Biological Studies

1. Cell Culture

HeLa cells were cultured in 'complete media' consisting of Dulbecco's modified Eagle medium (DMEM) supplemented with L-glutamine (4 mM), 10 % fetal bovine serum (FBS), and antibiotics (penicillin and streptomycin, 100 units/mL). Phenol red-free media was prepared using Fluorobrite™ DMEM using the same supplements. Cell culture was performed in a SteriCult 200 (Hucoa-Erloss) incubator at 5 % CO_2 atmosphere at 37 °C. To culture and plate the cells, the cells were washed with PBS, detached with trypsin/EDTA (0.25 % trypsin, 1 mM in PBS), diluted in DMEM, counted, then further diluted with DMEM to the appropriate concentration.

2. Cell Viability Assay

Cell viability was assessed using the MTT assay. HeLa cells were plated in a 96-well plate at 5,000 cells per well and allowed to grow to ~70% confluency overnight. Cells were incubated for 24 h with solutions of **P(DMA-co-CMACPT)** (6.75 - 250 µg/mL), or camptothecin (0.375 µM - 15 µM). The cell media was replaced with 100 µL of 3-(4,5-dimethylthiazol-2-yl)-2,5-diphenyltetrazolium bromide (MTT) solution (1 mg/mL) in PBS and the cells incubated for a further 3 h at 37 °C. After incubation, the resulting formazan crystals were dissolved by adding 100 µL of MTT solubilisation solution (10 % Triton-X 100 in 0.1 N HCl in isopropanol). The absorbance of the 96-well plates was read on a BioTek HT Synergy multimode reader at 570 nm using the Microplate manager 4.0 software, and the results compared to untreated (control) cells.

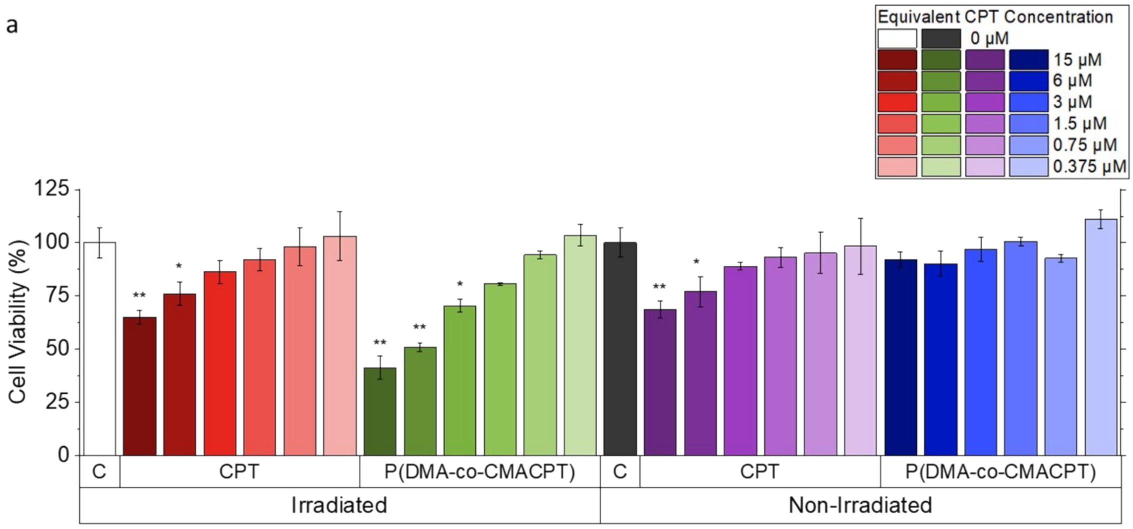

Figure S9. Cell viability of HeLa cells (MTT assay) following exposure to varying concentrations of P(DMA-co-CMACPT) (6.75- 250 μg/mL) and equivalent concentrations of camptothecin (0.375 μM to 15 μM) kept either in the dark or following a 2-minute 365 nm irradiation. a) 2 h post-treatment; b) 24 h post-treatment. Values are mean ± SD, n = 3, ** = $p \leq 0.01$, *** = $p \leq 0.001$, **** = $p \leq 0.0001$.

3. Intra-Cellular Drug Release Studies

Cells were plated in 96 well plates (10,000 cell per well density), and allowed to grow to ~60 % confluency overnight. Cells were then incubated with the polymer **P(DMA-co-CMACPT)** in 200 μL complete media for 24 hours at concentrations of 25 μg/mL, 100 μg/mL, and 250 μg/mL (corresponding to concentrations of camptothecin of 1.5 μM, 6 μM, and 15 μM, respectively). The polymer solutions were then removed, the cells were washed with phenol red-free media and 200 μL

of phenol red-free media was added to each well. Cells were then either placed in the dark or irradiated for 2 minutes in a UVP CL-1000 Crosslinker (Analytik Jena GmbH, Jena, Germany) equipped with five 8W UV-A tubes (F8T5/BL368; λ_{max} = 365 nm; 5 x 8W, 2 mW cm^{-2}). MTT assays were performed 2, 4 or 24 h later.

4. Live Cell Microscopy

HeLa cells incubated with **P(DMA-co-CMACPT)** were prepared the same as indicated in the cell viability experiment section, and were imaged using the brightfield of a Leica fluorescence microscope under a 10x objective.

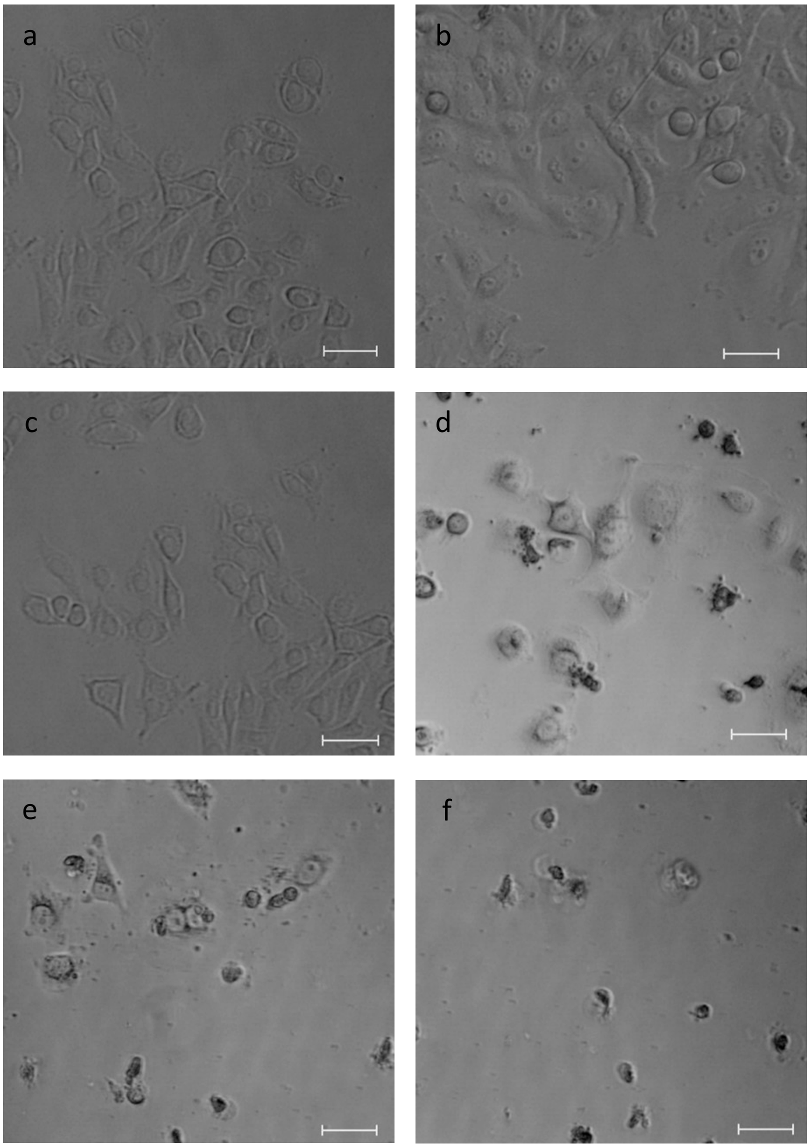

Figure S10. HeLa cell morphology under bright field microscopy when incubated with **P(DMA-co-CMACPT)**. Scale bar = 50 µm. a) control cells, non-irradiated. b) control cells, 2 mins 365 nm irradiation. c) 250 µg/mL **P(DMA-co-CMACPT)**, non-irradiated. d) 25 µg/mL **P(DMA-co-CMACPT)**, 2 mins 365 nm irradiation. e) 100 µg/mL **P(DMA-co-CMACPT)**, 2 mins 365 nm irradiation. f) 250 µg/mL **P(DMA-co-CMACPT)**, 2 mins 365 nm irradiation. Cells show dark round morphologies with uneven borders and internal bubble-like structures, indicating different states of cell-death post-irradiation.

IV. Investigations of the behaviour of CMA under irradiation

The ester bond at the 7-position of the coumarin monomer **CMA** was reported by Auzély-Velty, Woisel *et al.* to be cleaved under UV light irradiation (365 nm) or under two-photon excitation (~700 nm) leading to release of methacrylic acid (or the acid on the polymer backbone).[6] To investigate this unusual claim of photosensitivity in 7-position, and further control the behaviour of our polymer P(DMA-CMACPT) and its by-product under irradiation, we performed UV irradiation experiments on the monomer **CMA**, and on a water-soluble random co-polymer of DMA and CMA.

1. Synthesis of the P(DMA-co-CMA) random copolymer

P(DMA-co-CMA)

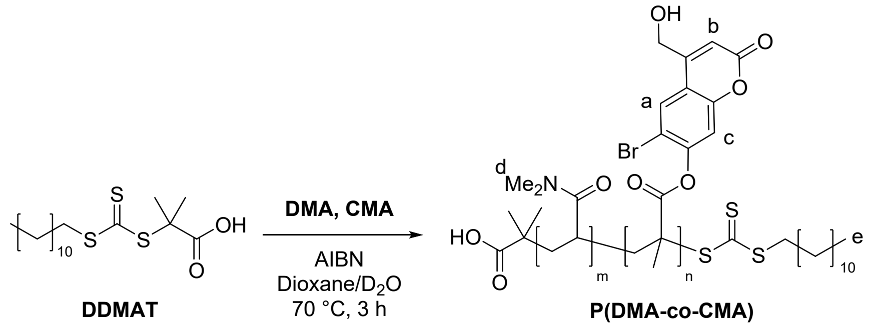

The random copolymer P(DMA-CMA) was synthesized according to the procedure described in section 1.3 of the ESI, from the coumarin monomer **CMA** (17 mg) and DMA (100 µL), and using **DDMAT** (3 mg) as the RAFT agent and AIBN (41 µg) as the initiator. The polymerisation was quenched after 3 hours with liquid nitrogen under air, and the polymer was purified by repeated precipitation in Et_2O and resuspension in MeOH (× 3).

^{1}H NMR (601 MHz, D_2O) δ (ppm) 8.25 – 7.76 (H_a), 7.71 – 7.20 (H_c), 6.76 – 6.41 (H_b), 3.54 – 2.29 (H_d), 1.00 – 0.81 (H_e). **Mw (GPC):** 9.6 kDa. **PDI:** 1.42.

2. Irradiation of the CMA monomer

The behaviour of the **CMA** monomer under 365 nm irradiation was first evaluated using HPLC (Figure S10). A 357 µM solution of **CMA** in MeCN:H_2O (8:2, *v*:*v*, 0.1 % formic acid buffer) was added to a quartz cuvette, and irradiated at 365 nm under stirring for a total of 60 minutes taking aliquots (50

µL) at regular time points. The aliquots were eluted on an HPLC with detection at 282 nm. The changes were assessed by measuring the area of the peak related to **CMA** (4.65 minutes). Photocleavage at the 7-position would be evidenced by the appearance of a peak related to compound **4** (3.20 min), however, after 60 minutes, the area of the peak of CMA remained constant (Figure S10, a), and no new peaks appeared (Figure S10, b and c).

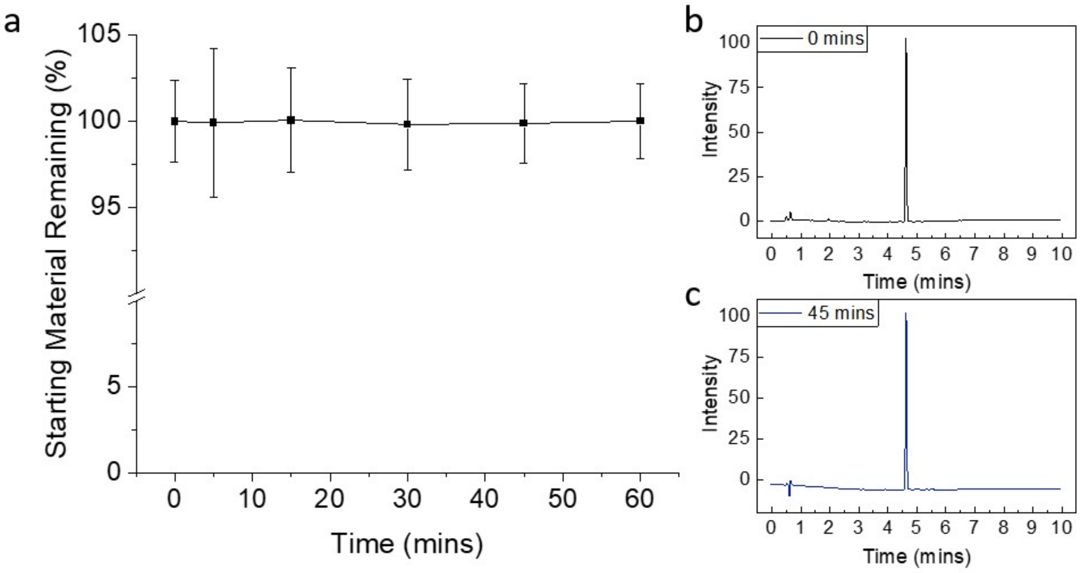

Figure S11. a) Percentage of **CMA** remaining following 365 nm irradiation over 60 minutes. Samples were analysed via HPLC (282 nm detection). SD was determined from triplicate measurements; b) HPLC trace of **CMA** at t = 0; c) HPLC trace of **CMA** after 45 minutes of 365 nm irradiation.

To further validate these results with structural information, an irradiation experiment was followed by ^{1}H NMR. A 2 mg/mL solution of **CMA** in DMSO-d_6 was added to a quartz cuvette, and irradiated at 365 nm under stirring for a total of 180 minutes. This long irradiation time was selected to thoroughly assess the stability of **CMA** under irradiation, and validate that the absence of cleavage is not due to slow kinetics. ^{1}H NMR spectra were acquired before and after the irradiation, showing no change in the signals (Figure S11, a and b).

Note: the water content in the sample increased during the course of the experiment due to the hygroscopicity of the DMSO-d_6, which is the cause of the proton of the hydroxyl group no longer being visible following irradiation.

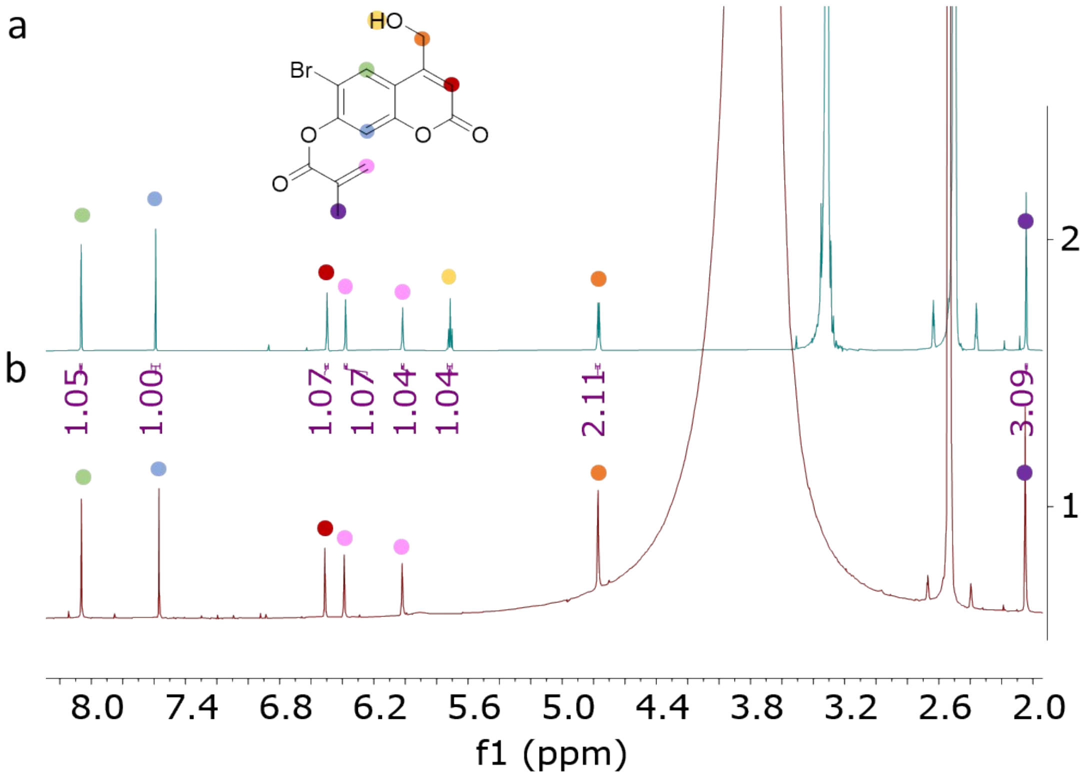

Figure S12. ^1H NMR spectra of CMA in DMSO-d_6 a) prior to irradiation, and b) after 180 minutes of 365 nm irradiation.

3. Irradiation of the P(DMA-co-CMA) random polymer: NMR follow-up

The water-soluble random copolymer **P(DMA-co-CMA)** was irradiated in aqueous conditions and followed by ^1H NMR to verify that the behaviour of the coumarin unit did not change after polymerisation of the methacrylate. This experiment was performed to in conditions similar to those described by Auzély-Velty, Woisel *et al.*, in which a hydrophilic polymer of **CMA** was reported to be light cleavable in aqueous conditions.[6] Cleavage at the 7-position would here imply that the side chains of **P(DMA-co-CMA)** would be released in the form of carboxylic acids, with the by-product **4** being water soluble. The intensity of the broad ^1H NMR signals (6.5 - 8.5 ppm) corresponding to the coumarin along the polymer chain would be expected to decrease, while narrow peaks corresponding to the released coumarin **4** should appear.

A 2 mg/ml solution of **P(DMA-co-CMA)** in D_2O was added to a quartz cuvette, and irradiated at 365 nm under stirring for a total of 30 minutes. ^1H NMR spectra were acquired before and after the irradiation (Figure S12). After 30 minutes under 365 nm light, no change in the ^1H NMR signals could

be seen, and the integrations of the peaks remained constant (within the 5 % error margin of NMR spectra), indicating that no cleavage occurred.

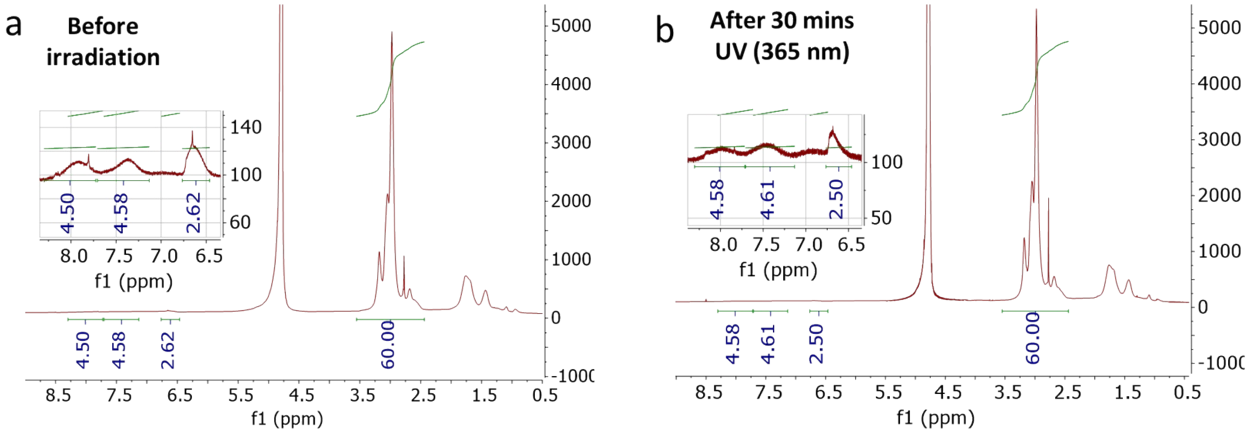

	Range	Normalized	Absolute
1	8.30 .. 7.72	4.50	379903.86
2	7.71 .. 7.13	4.58	387047.78
3	6.76 .. 6.46	2.62	221229.54
4	3.55 .. 2.44	60.00	5065488.82

	Range	Normalized	Absolute
1	8.30 .. 7.72	4.58	404635.48
2	7.71 .. 7.13	4.61	406468.38
3	6.76 .. 6.46	2.50	220341.93
4	3.55 .. 2.44	60.00	5295567.79

Figure S13. ^{1}H NMR spectra of **P(DMA-co-CMA)** in D_2O. Peaks between 2.44-3.55 ppm correspond to the backbone of the poly(*N*,*N*-dimethylacrylamide), integrated to 60 to represent 6 protons, and signals at 6.5 - 8.5 ppm correspond to the polymerised CMA units. a) Before irradiation, showing the integration of the coumarin peaks with respect to the PDMA backbone. b) After 30 minutes of 365 nm irradiation, showing the same ratios of integration, indicating the absence of cleavage. Tables indicated the range of chemical shift selected for comparable determination of the integrations, with normalized and absolute integration values given for each signal.

4. Irradiation of the P(DMA-co-CMA) random polymer: absorption follow-up

To further confirm the absence of photocleavage in polymerised CMA, absorption spectra of the random copolymer **P(DMA-co-CMA)** before and after irradiation were acquired. Thanks to a free phenol at the 7-position, the absorption spectrum of coumarin **4** is strongly red-shifted compared to that of the methacrylate ester **CMA** (Figure S13, a and b; note that spectra were acquired in MeOH to ensure solubility of **CMA**). If a photo-cleavage occurred, a red-shift in the absorbance peak of the polymer solution (from ~320 nm to ~ 365 nm) should therefore be observed. This red-shift should be

even more pronounced and noticeable in neutral aqueous buffer, were compound **4** exists primarily under its phenolate form thanks to the low pKa of the phenol in BHC.[7]

A 1 mg/ml solution of **P(DMA-co-CMA)** in PBS was added to a quartz cuvette, and irradiated at 365 nm under stirring for a total of 30 minutes. Absorption spectra were acquired before and after the irradiation (Figure S13, c), showing no change in the signals. This further confirms photo-stability of the CMA units, and the absence of cleavage from the polymer backbone. On the contrary, the CMA monomer exhibits exceptional photo-stability, which indicates that the by-product of the photolysis of our P(DMA-co-CMACPT) is not degraded by light.

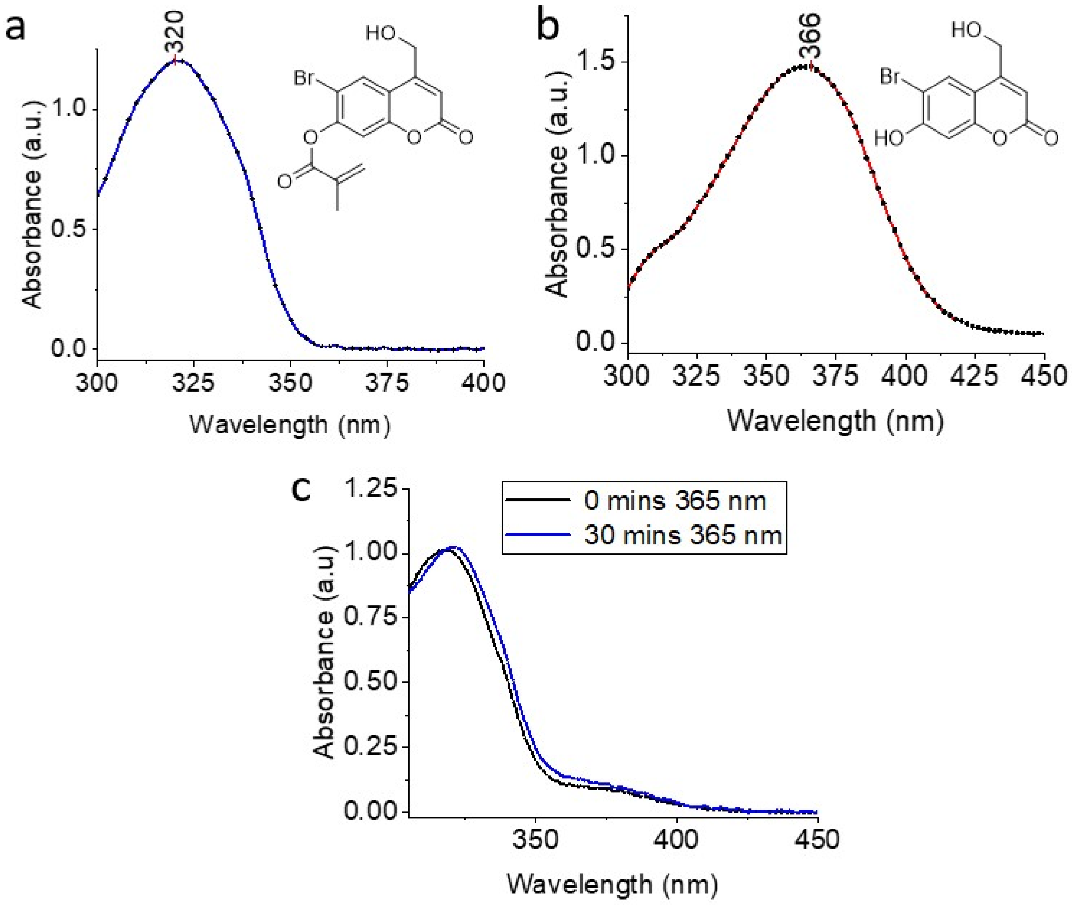

Figure S14. a) Absorption spectrum of **CMA** in MeOH. b) Absorption spectrum of **4** in MeOH. c) Absorption spectra of P(DMA-co-CMA) before and after 30 minutes of 365 nm irradiation.

V. NMR, MS and HPLC data

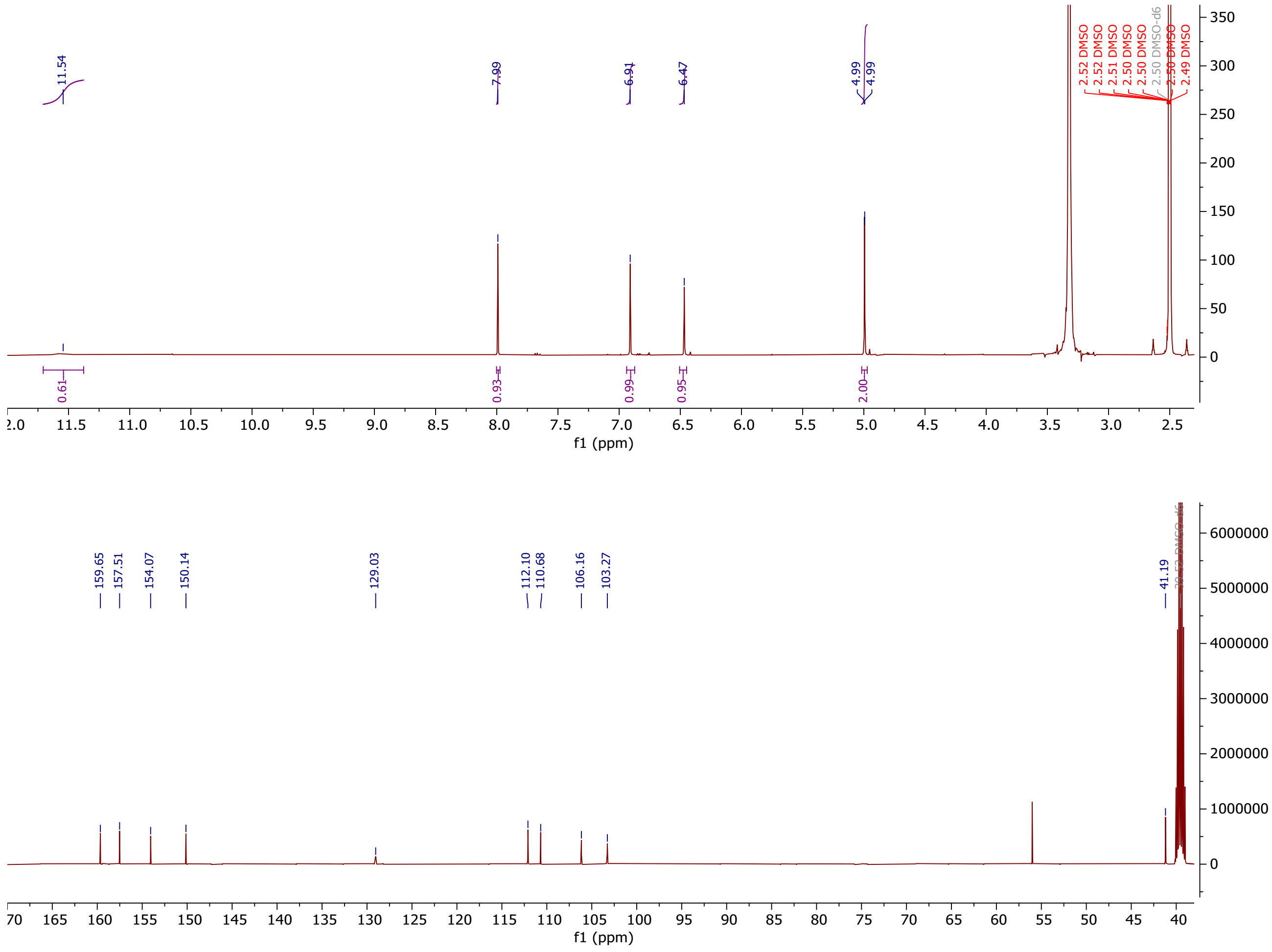

Figure S15. ^{1}H (top) and ^{13}C NMR (bottom) spectra of compound **3**, recorded at 500 MHz and 126 MHz respectively, in DMSO-d_6.

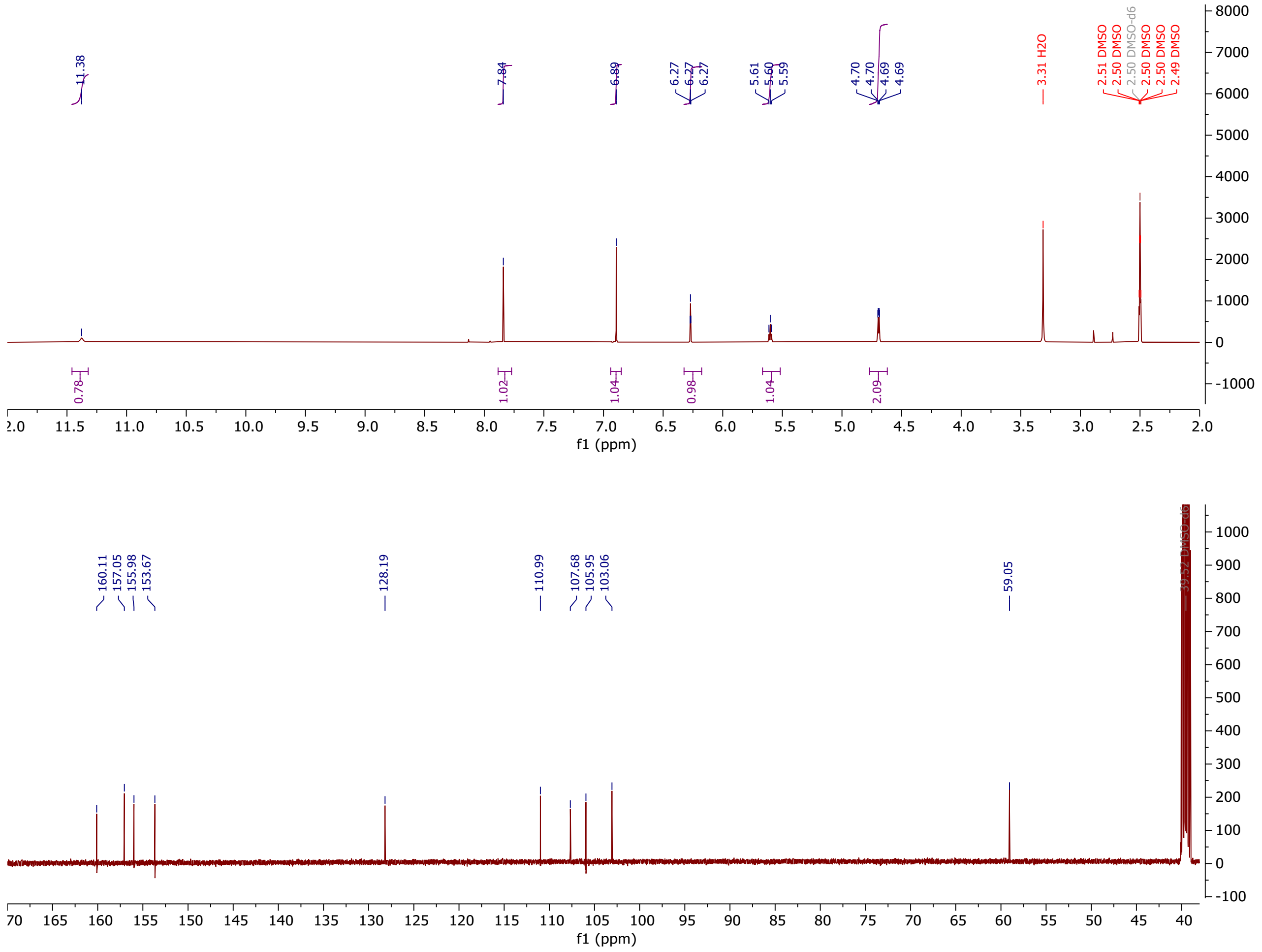

Figure S16. ^{1}H (top) and ^{13}C NMR (bottom) spectra of compound **4**, recorded at 500 MHz and 126 MHz respectively, in DMSO-d_6.

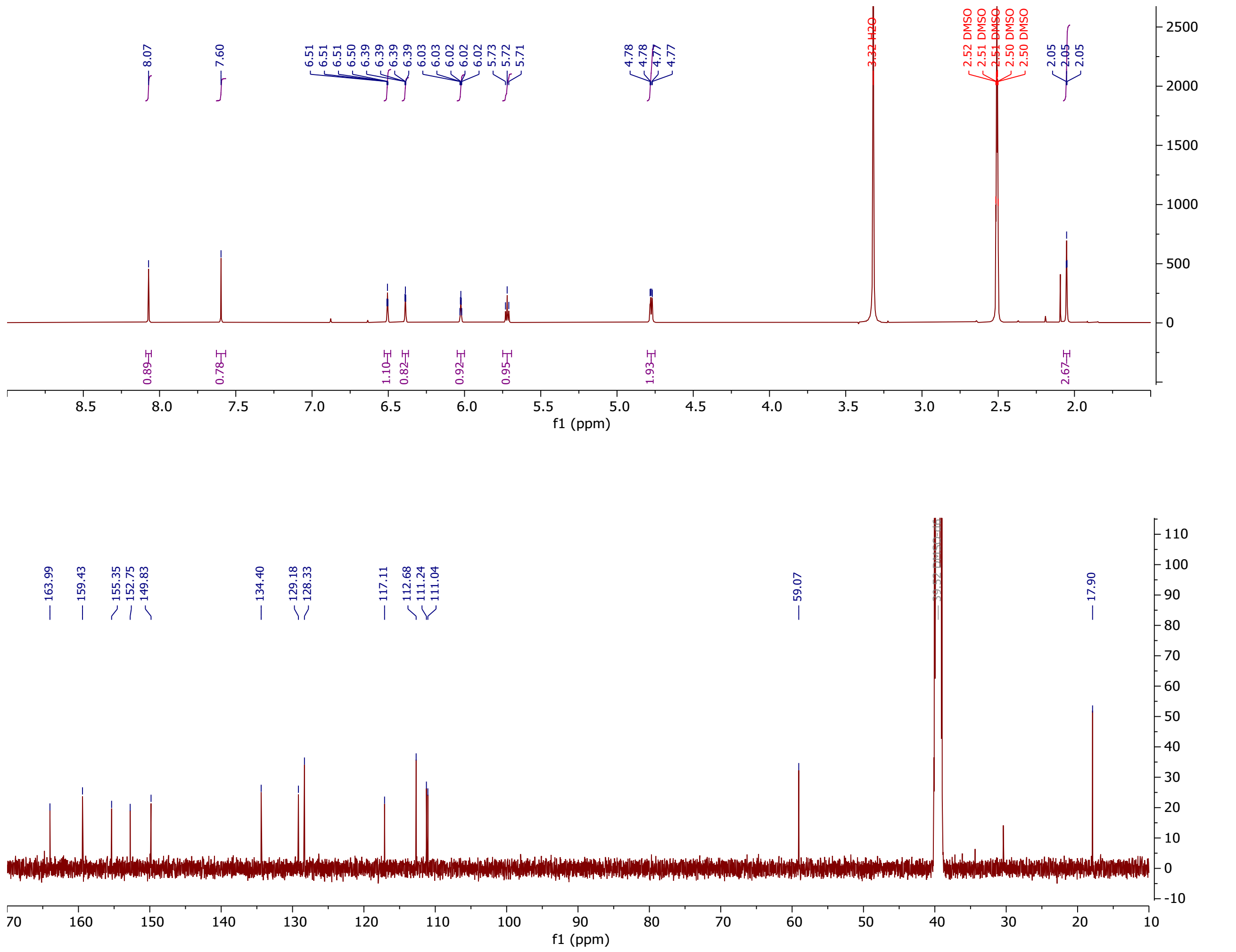

Figure S17. ^{1}H (top) and ^{13}C NMR (bottom) spectra of **CMA**, recorded at 500 MHz and 126 MHz respectively, in DMSO-d_6.

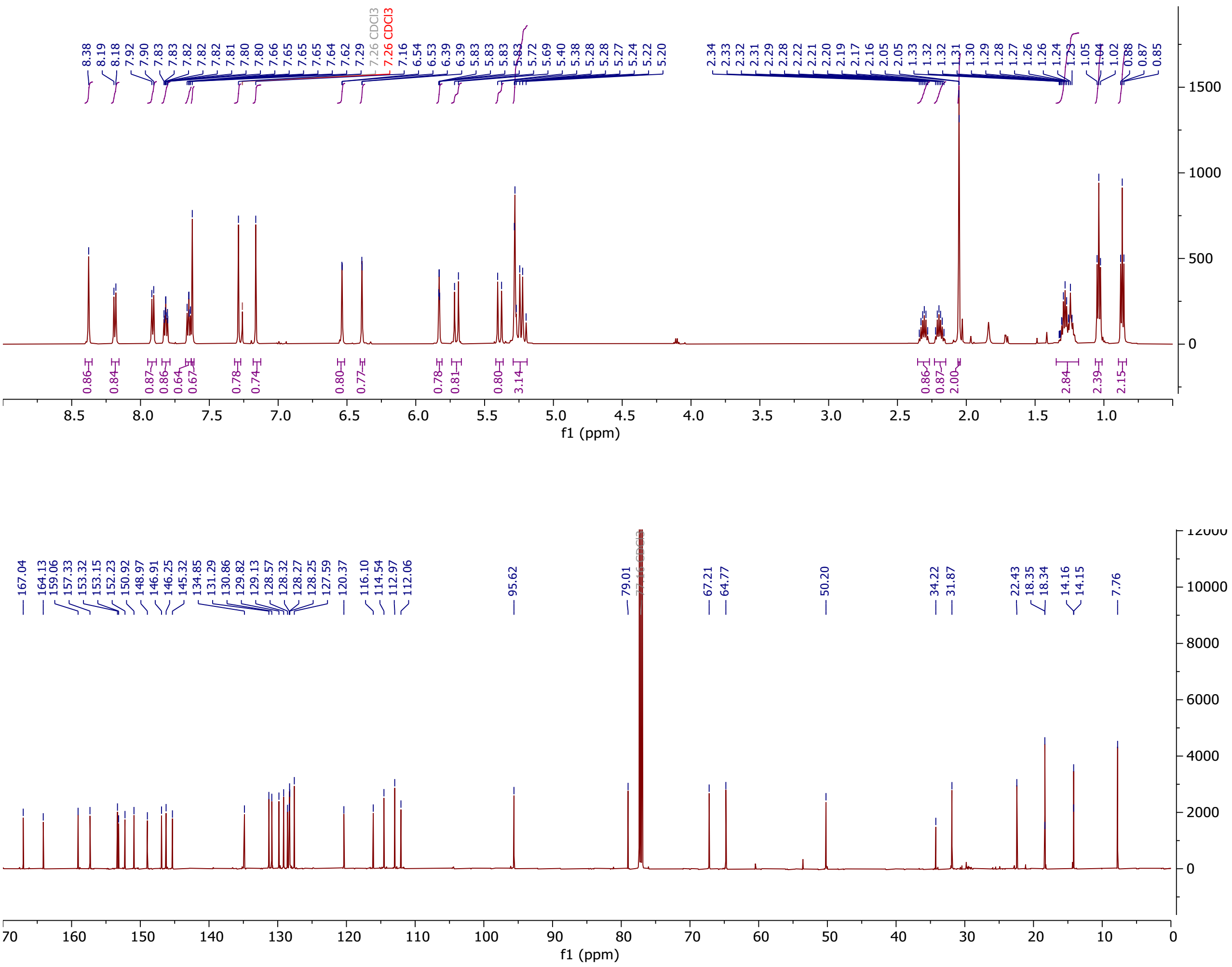

Figure S18. ^{1}H (top) and ^{13}C NMR (bottom) spectra of **CMACPT**, recorded at 500 MHz and 126 MHz respectively, in $CDCl_3$.

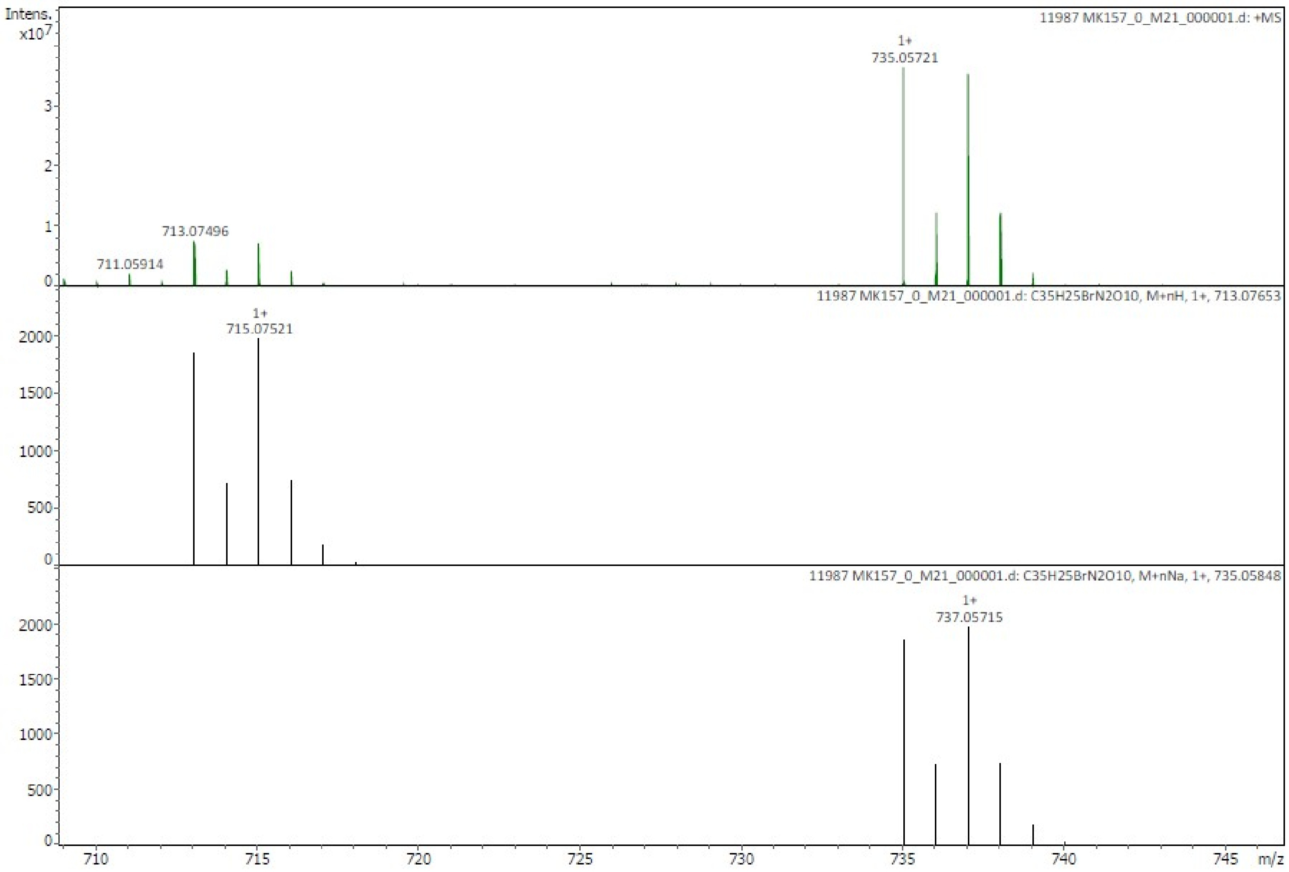

Figure S19. HRMS (ESI) spectrum for compound **CMACPT**.

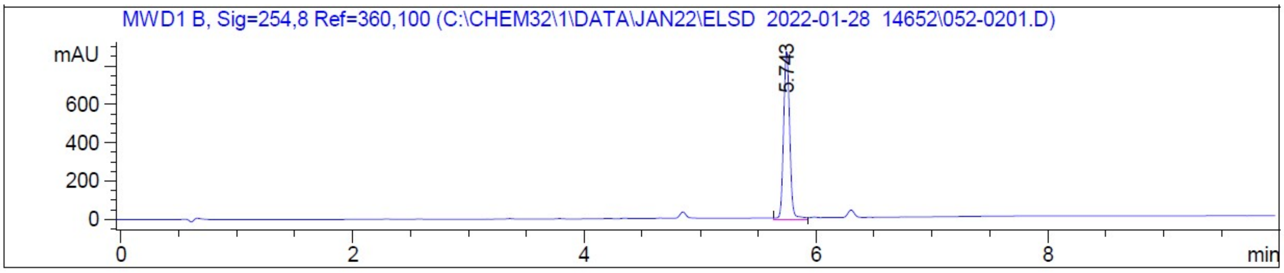

Figure S20. HPLC trace (254 nm detection) for compound **CMACPT**.

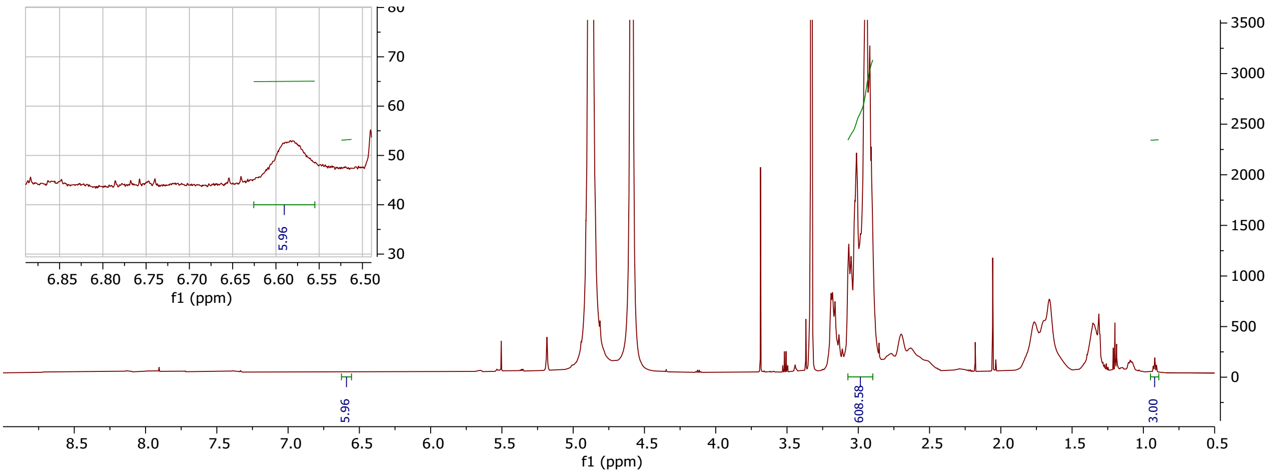

Figure S21. ¹H NMR spectrum of **P(DMA-co-CMACPT)**, recorded at 600 MHz in CD_3OD. The signals at 2.85 – 3.10 ppm (PDMA units) and at 6.62 – 6.55 ppm (coumarin units) were integrated with respect to the triplet at 0.92 ppm (terminal CH_3 on the RAFT agent) to determine the size of the polymer.

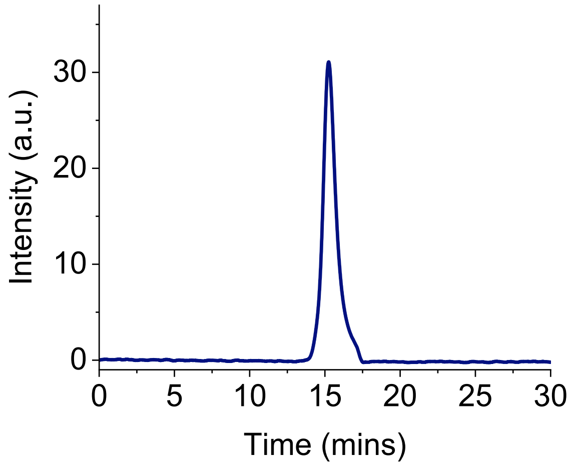

Figure S22. GPC trace (282 nm detection) of the 12 kDa polymer **P(DMA-co-CMACPT)**.

VI. Additional references

1 S.-I. Murahashi, D. Zhang, H. Iida, T. Miyawaki, M. Uenaka, K. Murano and K. Meguro, *Chem. Commun.*, 2014, **50**, 10295–10298.

2 C. G. Hatchard, C. A. Parker and E. J. Bowen, *Proceedings of the Royal Society of London. Series A. Mathematical and Physical Sciences*, 1997, **235**, 518–536.

3 M. Montalti, A. Credi, L. Prodi and M. T. Gandolfi, in *Handbook of Photochemistry*, CRC Press, 3rd edn., 2006.

4 N. Adarsh, R. R. Avirah and D. Ramaiah, *Org. Lett.*, 2010, **12**, 5720–5723.

5 S. B. Partanen, P. R. Erickson, D. E. Latch, K. J. Moor and K. McNeill, *Environ. Sci. Technol.*, 2020, **54**, 3316–3324.

6 C. Benoit, S. Talitha, F. David, S. Michel, S.-J. Anna, A.-V. Rachel and W. Patrice, *Polym. Chem.*, 2017, **8**, 4512–4519.

7 T. Furuta, S. S.-H. Wang, J. L. Dantzker, T. M. Dore, W. J. Bybee, E. M. Callaway, W. Denk and R. Y. Tsien, *PNAS*, 1999, **96**, 1193–1200.
